# Genome-wide characterization of the sunflower kinome: classification, evolutionary analysis and expression patterns under different stresses

**DOI:** 10.3389/fpls.2024.1450936

**Published:** 2024-12-02

**Authors:** Ningning Yan, Shuqing Yang, Haoyu Chao, Wenbing Zhang, Jian Zhang, Ming Chen, Jun Zhao

**Affiliations:** ^1^ College of Horticultrue and Plant Protection, Inner Mongolia Agricultural University, Hohhot, China; ^2^ College of Agriculture, Tarim University, Alaer, China; ^3^ Institute of Crop Science and Zhejiang Key Laboratory of Crop Germplasm, Zhejiang University, Hangzhou, China

**Keywords:** protein kinase, biotic stress, sunflower, abiotic stress, transcriptome

## Abstract

Protein kinases play a significant role in plant responses to biotic and abiotic stresses, as well as in growth and development. While the kinome has been extensively investigated in crops such as *Arabidopsis thaliana*, soybean, common bean, and cotton, studies on protein kinases in sunflower remain limited. Our objective is to explore protein kinases in sunflower to bridge the research gap and enhance the understanding of their functions. We identified a total of 2,583 protein kinases from sunflower, which were classified into 22 families and 121 subfamilies. By comparing the subfamily members between sunflower and other species, we found that three subfamilies in sunflower—RLK-Pelle_CrRLK1L-1, RLK-Pelle_SD-2b, and RLK-Pelle_WAK—had undergone significant expansion. We then investigated the chromosomal distribution, molecular weight, isoelectric point, transmembrane domain, signal peptide, and structural and evolutionary diversity of the protein kinases. Through these studies, we have obtained a basic understanding of protein kinases in sunflower. To investigate the role of protein kinases in sunflower’s response to biotic and abiotic stresses, we obtained 534 transcriptome datasets from various research groups, covering eight types of abiotic stress and two types of biotic stress. For the first time, we overcame the batch effects in the data and utilized a gene scoring system developed by our lab to perform a comprehensive analysis of multiple transcriptome datasets from different research groups. Ultimately, 73 key protein kinases were identified from numerous candidates, and functional annotation revealed that they are key members of signaling pathways such as ABA, MAPK, and SOS, actively participating in sunflower’s response to biotic and abiotic stresses. In summary, through the exploration of protein kinases in sunflower, we have filled the gap in protein kinase research and provided a substantial amount of foundational data. By using the new scoring method to eliminate batch effects between transcriptome datasets, we achieved the first comprehensive analysis of large-scale transcriptome data. This method allows for a more thorough and detailed identification of key protein kinases that are widely regulated under various stress conditions, providing numerous candidate genes for sunflower stress resistance research.

## 1 Introduction

Plants face constant challenges from adverse abiotic stressors such as drought, heat, cold, salt, and flooding. Additionally, they encounter biotic stresses caused by fungi, bacteria, nematodes, insects, herbivores, and weeds. The occurrence of these stresses can alter the physiological and biochemical responses of plants (Nawaz et al., 2023), negatively affecting their growth and development. To cope with these threats, plants have evolved sophisticated mechanisms. Various physiological and biochemical responses to stress, including stomatal closure, calcium influx, changes in osmotic pressure, oxidative burst, and hypersensitivity reactions, help ensure normal growth and development (Fang and Xiong, 2015). In the process of plant resistance to various stresses, protein phosphorylation serves multiple functions, including stress recognition and signal transduction. Protein phosphorylation plays a crucial role in regulating nearly all cellular processes and enables cells to rapidly respond to both abiotic and biotic stresses (Gong et al., 2020; Defalco and Zipfel, 2021; Escocard De Azevedo Manhães et al., 2021; Mishra et al., 2021; Soltabayeva et al., 2022).

In 1955, Fischer and Krebs made their discoveries in the field of reversible phosphorylation, involving the attachment or detachment of phosphate groups to cellular proteins. Their significant contributions in this area earned them the prestigious Nobel Prize in Physiology or Medicine in 1992 (Fischer and Krebs, 1955; Krebs and Fischer, 1955). Protein phosphorylation is a common and essential post-translational modification of proteins. In this process, protein kinases play a key role by catalyzing phosphorylation reactions, transferring the terminal γ-phosphate of ATP to specific amino acid residues (serine, threonine, and tyrosine) on the substrate protein (Cohen, 2002). This phosphorylation event induces changes in the protein’s structural conformation, ultimately impacting its function (Holzberg et al., 2002). Phosphorylation events act as switches that can activate or deactivate proteins, thereby initiating or terminating signal transduction. Signal transduction is integral to various processes such as plant immunity, response to environmental stresses, and regulation of growth. Plant stresses typically lead to oxidative stress, water imbalance, membrane and cell wall damage, and osmotic pressure changes. Through the recognition and transmission of relevant signals, protein kinases facilitate adaptive adjustments in plants at both physiological and biochemical levels (Hua et al., 2012; Ding et al., 2015; Zhou et al., 2018). For instance, the protein kinase CaCIPK13 enhances pepper’s ability to withstand low temperatures (Ma et al., 2022), while the protein kinase ZmCDPK7 improves maize’s tolerance to high temperatures (Zhao et al., 2021). In the presence of invading pathogens like bacteria, fungi, and viruses, pattern recognition receptors located on the cell membrane, usually kinases or kinase partners, initiate signal transmission to the nucleus, leading to the activation of disease-related protein expression (Wang et al., 2015; Labbé et al., 2019). For example, when the calcium-dependent protein kinase TaCDPK27 is silenced, wheat’s resistance to powdery mildew is enhanced (Yue et al., 2023). In addition to stress responses, protein kinases also play a significant role in plant hormone signaling and growth regulation (Wan et al., 2008).

Apart from studying individual protein kinases involved in plant stress responses, numerous researchers have conducted comprehensive investigations on protein kinases across various plant species. For example, Monika (Zulawski et al., 2014) discovered 942 protein kinases in *Arabidopsis*, constituting approximately 2.7% of its protein-coding genes. Similarly, Liu (Liu et al., 2015) identified 2166 protein kinases in soybean, accounting for approximately 4.7% of its protein-coding genes. Moreover, studies on the kinome have been carried out in other plants such as tomato (Singh et al., 2014), maize (Wei et al., 2014), *Brassica napus* (Gill et al., 2017), pineapple (Zhu et al., 2018a), grapevine (Zhu et al., 2018b), *Gossypium* spp (Yan et al., 2018)., wild strawberry (Liu et al., 2020), rubber tree (Santos et al., 2022), and cowpea (Ferreira-Neto et al., 2021). Sunflower, a significant oil crop, is cultivated in many countries worldwide, including Russia, Ukraine, Argentina, Romania, Tanzania, China, France, and Turkey. From 2014 to 2018, sunflower seed production ranked third among oilseed crops globally, contributing approximately 9% to total oilseed production, following soybeans and rapeseed. Sunflower currently faces various challenges, including abiotic stresses like global warming and water resource depletion, as well as biotic stresses like pathogens and weeds. In light of these threats, developing cultivars with resistance to diseases, drought, heat, and improved quality traits represents a highly cost-effective solution. Protein kinases are crucial in sunflower’s response to both abiotic and biotic stresses. Exploring the role of protein kinases in sunflower can provide a strong theoretical foundation for breeding superior sunflower varieties. The kinome of sunflower remains unexplored, but valuable resources now make the investigation of sunflower protein kinases possible. In 2017, Hélène Badouin published the genome of the inbred line XRQ, which included 17 pseudomolecules and 1,509 unanchored contigs (Badouin et al., 2017). Additionally, numerous high-quality transcriptome datasets have been made available on NCBI, offering insights into sunflower genes involved in responding to drought, PEG6000, cold, heat, and biotic stress. By combining genomic and transcriptome data, we can identify key protein kinases responsible for sunflower’s response to both abiotic and biotic stresses.

The objective of this study was to identify, classify, and catalog the protein kinases (PKs) in sunflower. To achieve this, we identified 2,583 loci encoding for sunflower PKs (HaPKs) within the reference genome. Subsequently, we performed phylogenetic analysis and used iTak classification to categorize the genes encoding HaPKs. Additionally, we analyzed the properties of all HaPKs, including molecular weight (MW) and isoelectric point (pI). The study also investigated the mechanisms underlying the expansion of HaPKs in the sunflower genome and conducted synteny analysis among sunflower species. Furthermore, we explored the role of HaPKs in sunflower’s response to both biotic and abiotic stresses by analyzing available transcriptome data. From this analysis, we identified 73 significant HaPKs for further investigation of their tissue-specific expression patterns and responses to various hormone treatments.

## 2 Materials and methods

### 2.1 Identification and classification of sunflower protein kinases

We obtained the sunflower genome, proteome, and annotation files from the INRAE Sunflower Bioinformatics Resources (https://www.heliagene.org/HanXRQr2.0-SUNRISE/). To identify PKs, we downloaded Hidden Markov models (HMMs) for the Pkinase (PF00069) and Pkinase_Tyr (PF07714) families (Finn et al., 2010) from the Pfam database (https://www.ebi.ac.uk/interpro/entry/pfam/#table). Using HMMER v.3.2.1 (Finn et al., 2011) in the TBtools software v1.113 (Chen et al., 2020), we aligned the protein sequences to each HMM profile with an E-value cut-off of 1.0e−5 to identify potential PKs. These potential PKs were then verified using InterProScan-5.60-92.0 against the Pfam database, and we just retained PKs that contained at least one kinase domain. We utilized the iTak software (Zheng et al., 2016) to categorize the retained PKs into families, with the potential to identify up to 150 kinase families. To confirm the classification, we performed phylogenetic analysis. Specifically, we aligned the full-length amino acid sequences of the candidate PKs using ClustalW 2.1 (Larkin et al., 2007) and constructed a phylogenetic tree with FastTree 2.1.11, using the maximum likelihood method (Jones-Taylor-Thornton (JTT) model) with a bootstrap value of 1000 (Price and Dehal, 2010). The generated tree was then visualized with MEGA11 (Tamura and Stecher, 2021). To be considered typical PKs, the putative PKs had to appear in the same family according to the results of the iTAK software and the phylogenetic tree clade.

### 2.2 Kinase characterization

In order to provide comprehensive information about each HaPK, we investigated the following characteristics: (a) the presence of transmembrane domains and N-terminal signal peptides, which were determined using DeepTMHMM v.1.0.24 (Hallgren et al., 2022) and Signal v.5.0 (Almagro Armenteros et al., 2019); (b) the chromosome location and intron/exon organization, as annotated in the GFF files; (c) the molecular weight (MW) and isoelectric point (pI), which were calculated using ExPASy (Duvaud et al., 2021) (https://web.expasy.org/compute_pi/); (d) the prediction of protein domains using the Web CD-search Tool (Lu et al., 2020)(https://www.ncbi.nlm.nih.gov/Structure/bwrpsb/bwrpsb.cgi). The results of domain predictions were visualized using TBtools software v1.113.

### 2.3 Duplication events and Ka and Ks calculation in the sunflower kinome

We employed the Multiple Collinearity Scan toolkit (MCScanX) (Wang et al., 2012) To analyze collinear blocks and classify duplication events among the HaPKs. The HaPKs were categorized into singletons, dispersed duplicates, proximal duplicates, tandem duplicates, and segmental/whole genome duplicates (WGD). The visualization of WGD and tandem duplication events were carried out using TBtools software (Chen et al., 2020). Additionally, we utilized the WGDI software (Sun et al., 2022) to predict and visualize whole genome duplication events. To further investigate the duplication events, we calculated the synonymous substitution (*Ks*) and nonsynonymous substitution (*Ka*) rates for WGD and tandem duplications. The dates of these duplication events were estimated using the Ks values with the equation T = Ks/2r, where r = 8.25×10^-9^ (Strasburg and Rieseberg, 2008).

### 2.4 RNA-seq expression pattern analysis

We retrieved public RNA-seq expression data of sunflower from the NCBI`s Sequence Read Archive (SRA) to explore the potential functions of HaPKs under multiple abiotic and biotic stress. The RNA-seq datasets in response to abiotic stress including cold, heat, salt, alkali, drought, flooding, PEG6000, low-nutrient coming from different projects were used (SRP326108, SRP355134, SRP162252, SRP294448, SRP392176, SRP092742) and the RNA-seq datasets containing samples from different biotic stresses were downloaded from the project SRP411503(infection by *Sclerotinia sclerotiorum*) and SRP173084 (parasitizing by *Orobanche cumana*). In the meantime, we assessed the expression patterns of PKs in various tissues (root, stem, leaf, bract, pollen, stamen, pistil, DF ovary, DF corolla, RF ovary, RF ligule) of sunflower under normal conditions. We also analyzed the expression profile of PKs of leaf and root under treatments with different phytohormones (ABA, SA, MeJA, IAA, BRAS, ACC, GA3 and kinetin). The RNA-seq raw data was acquired from a previous study (SRP092742).

The quality of RNA-seq raw data was assessed using software fastp. The resulting high-quality reads were mapped to sunflower reference genomes by software Star. The counts of expression genes were performed using software RSEM. The differentially expressed genes (DEGs) of sunflower under different abiotic and biotic stress were calculated using R package DESeq2. The expression fold change (FC) was derived for each of the treatments compared to the control. All statistical analyses and figures were performed using R project version 4.0.5.

In order to identify the most significant PKs in response to abiotic and biotic stress, we graded all the PKs across the entire sample set (**Supplementary Figure 1**). Initially, we classified the samples based on the specific type of stress they were subjected to, resulting in 10 distinct groups including cold, heat, salt, alkali, drought, flooding, PEG6000, low-nutrient, *Sclerotinia sclerotiorum* and *Orobanche cumana*. Subsequently, we proceeded to grade each individual PK. If a PK exhibited differential expression in the treatment group compared to the control group within a particular sample, it was assigned a score of 1. Finally, we computed the cumulative score for each PK across all samples, allowing us to determine their overall significance. On the other hand, due to varying sample sizes in each group, we resolved this discrepancy by calculating the average score for PKs within each group. By summing up these average scores, we obtained a total score that mitigated the issue of weight disparity. Then, we ranked the PKs based on their scores derived from the aforementioned two methods. To assess the significance of PKs comprehensively, we also incorporated the expression fold change as an important indicator. To achieve this, we rescored the PKs by assigning their respective fold change values instead of a fixed 1-point score. In summary, we employed four different methods to grade the PKs, ranking them accordingly in each method, resulting in the identification of the top 30 PKs in each set of results.

## 3 Results

### 3.1 Genome-wide identification and classification of HaPKs

Using HMMER v.3.2.1, we conducted a search for putative kinase domains in all 71,289 annotated sunflower proteins. This search yielded 2688 and 2650 putative kinases using HMM models of the typical kinase domain (PF00069 and PF07714), respectively. After merging the results of the two groups and removing duplicates, a total of 2,721 putative HaPKs were obtained. To further verify whether all the putative HaPKs contain kinase domains, we used the InterProScan-5.60-92.0 to analyze the domains they contain. Of the 2721 HaPKs, 2698 proteins (**Supplementary Table S1**) were found to contain at least one kinase domain.

**Figure 1:**
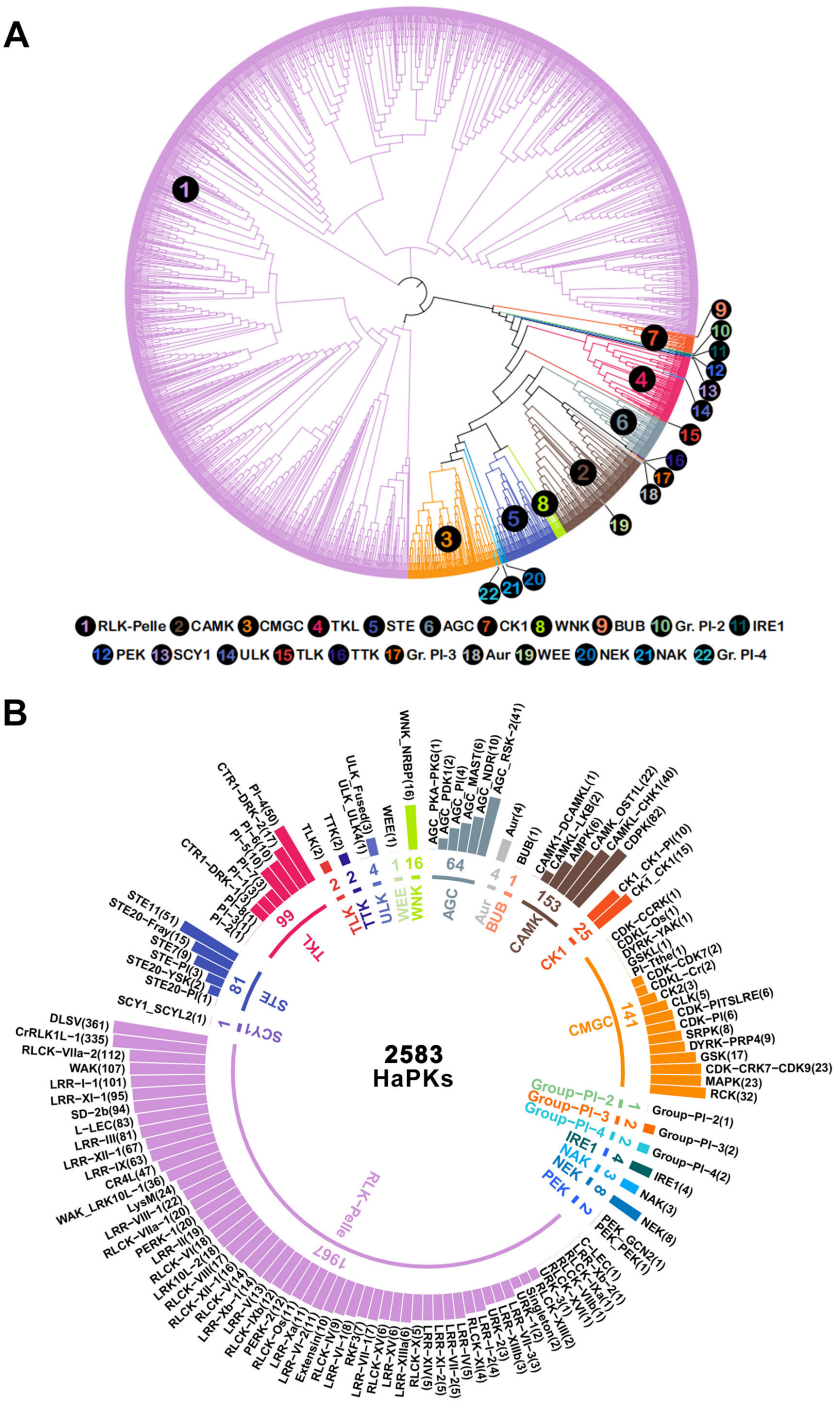
Classification and phylogenetic tree of sunflower protein kinases. **(A)** Protein kinases are divided into 22 families and 121 subfamilies. The inner circle shows the family name and number. The outer circle shows the names and numbers of the subfamilies, and the subfamilies are sorted by number and shown in a bar chart. **(B)** Analysis of protein kinase phylogenetic tree. The numbers correspond to 22 different families, the numbers inside the circle indicate the larger families, the numbers outside the circle correspond to the smaller families, and the color of the numbers corresponds to the color of the line in the evolutionary tree. Different families are represented in different colors in the two figures.

Upon analysis using the iTAK software, a total of 2,698 HaPKs were successfully classified into different families and subfamilies (**Supplementary Table S2**). To visualize the relationships of HaPKs in the families and subfamilies, a phylogenetic tree was constructed using the 2,698 proteins (**Figure 1A**; **Supplementary Figure 2**), 115 putative HaPKs exhibited divergences in the phylogeny and were consequently assigned to the unknown kinase (UNK) group. We ultimately obtained 2,583 HaPKs.

The 2,583 HaPKs were grouped into 22 families (**Figure 1B**; **Supplementary Tables S3**, **S4**), including AGC (PKs A, G and C), Aur (Aurora), BUB (budding uninhibited by benzimidazoles), CAMK (calcium and calmodulin-regulated kinases), CK1 (casein kinase 1), CMGC (cyclin-dependent, mitogen-activated, glycogen synthase, and CDC-like kinases), Group−Pl−2, Group−Pl−3, Group−Pl−4, IRE1 (plant-specific, inositol-requiring enzyme 1), NAK (NF-kB-activating kinase), NEK (NIMA-related kinase), PEK (Pancreatic eIF-2a kinase), RLK-Pelle (Receptor-like kinase), SCY1 (*Saccharomyces cerevisiae* Scy1 kinase), STE (Serine/threonine kinase), TKL (Tyrosine kinase-like kinase), TLK (Tousled-like kinases), TTK (Threonine/tyrosine kinase), ULK (Unc-51-like kinase), WEE (Wee1, Wee2, and Myt1 kinases), and WNK (with no lysine-K). The distribution of the family members is not uniform across all families. The family RLK-Pelle exhibited the highest number of members, accounting for 76.15% (1,967/2,583) of the HaPKs. Following RLK-Pelle, the families with the next highest member were CAMK (153) and CMGC (141). There are four families, BUB, Group-Pl-2, SCY1, and WEE, that each contain only one family member. Through further analysis, we divided the 22 families into 121 subfamilies. The larger the family, the more subfamilies it contains, while some smaller families were not further subdivided. The largest family, RLK-Pelle, was divided into 57 subfamilies, among which RLK-Pelle_DLSV and RLK-Pelle_CrRLK1L-1 are the two largest subfamilies, containing 361 and 335 family members, respectively, which is more than any other family contains.

**Figure 2:**
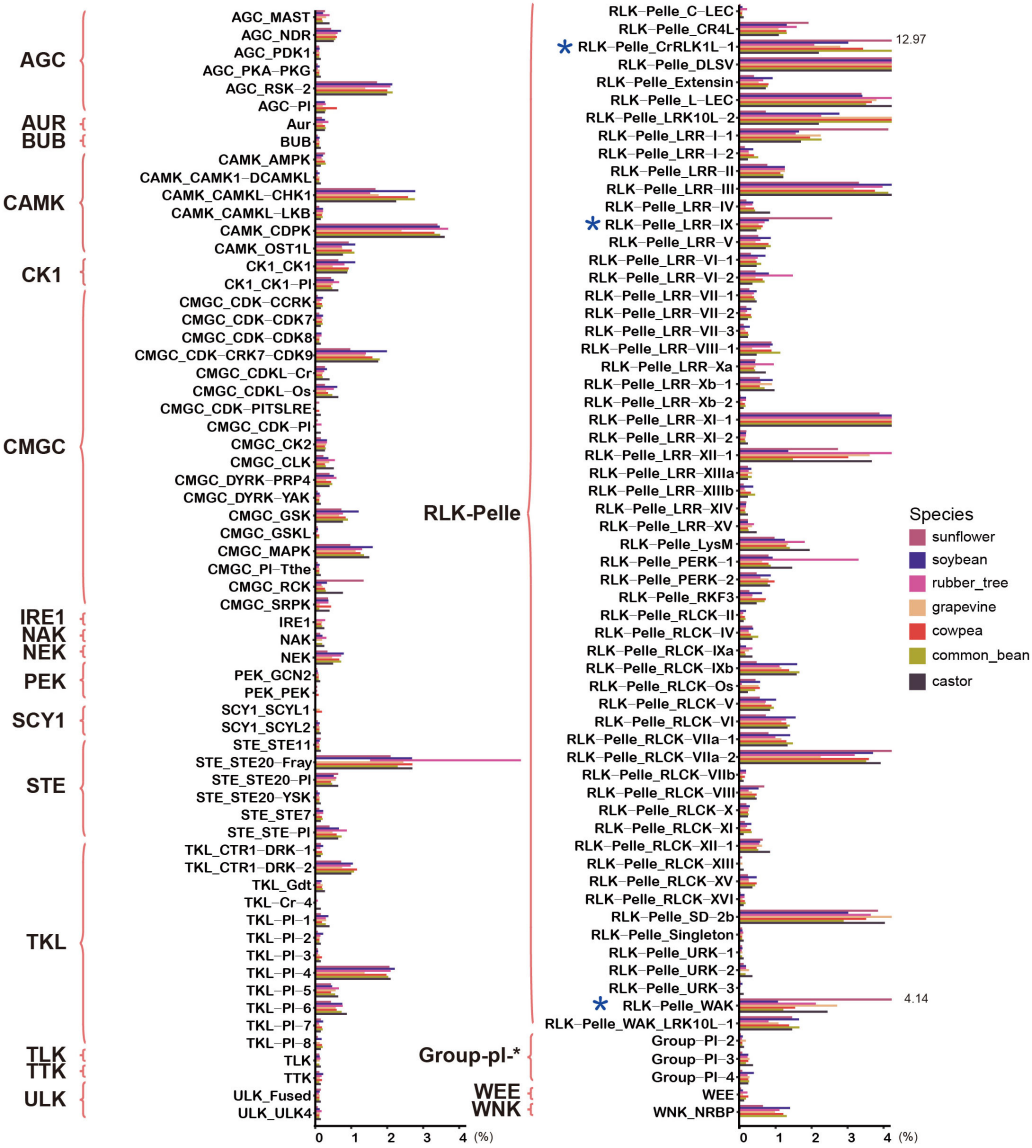
Comparison of the proportion of subfamily members in sunflower and other species. The bar chart represents the proportion of subfamily members in each species, with different species indicated by different colors. The chart also highlights families and subfamilies associated with HaPKs.

To compare the distribution characteristics of protein kinases in sunflower and other species, we obtained the protein kinases from six additional species. The number of protein kinases varied significantly among these six species, with the highest number found in the rubber tree and the lowest in castor. In sunflower, the number of protein kinases was second only to that of the rubber tree, indicating a large family that likely underwent numerous duplication events during evolution. To clarify the distribution of protein kinases in subfamilies across different species, we counted the number and proportion of subfamily members in the seven species (**Figure 2**; **Supplementary Table S5**). The analysis revealed that the size of most subfamilies was consistent across all species, such as the RLK-Pelle_DLSV subfamily, which was the largest in all species and had the same proportion. However, three subfamilies within the RLK-Pelle family in sunflower, namely RLK-Pelle_CrRLK-1, RLK-Pelle_LRR-IX, and RLK-Pelle_WAK, showed significant expansion. Their proportions in sunflower were notably higher than in other species. For example, RLK-Pelle_CrRLK-1 accounted for 12.97% in sunflower, while its highest proportion in other species was only 4.32%, and the lowest was 1.97%.

### 3.2 The properties of sunflower kinome

To gain insight into the sunflower kinome, we examined the domains of the 2,583 HaPKs (**Supplementary Table S6**). There are differences in the types and numbers of kinase domains contained in HaPKs. The majority of HaPKs (2394) contained only one kinase domain, while the remaining 189 contained 2-6 kinase domains (**Supplementary Table S7**). Among these, 55 HaPKs possessed two typical kinase domains of PF00069, while 61 HaPKs possessed 2-6 kinase domains of PF07714, with 73 HaPKs having both domains. Through statistical analysis of the subfamilies to which these 189 HaPKs belong, it was found that HaPKs containing multiple kinase domains are mainly concentrated in the subfamilies RLK-Pelle_CrRLK1L-1, RLK-Pelle_LRR-I-1, and AGC_RSK-2 (**Supplementary Table S7**). Among the 61 HaPKs containing multiple PF07714 domains, the RLK-Pelle_CrRLK1L-1 and RLK-Pelle_LRR-I-1 contain 36 and 9 members, respectively. Among the 55 HaPKs containing multiple PF00069 domains, the AGC_RSK-2, RLK-Pelle_CrRLK1L-1, and RLK-Pelle_LRR-I-1 contain 31, 6, and 4 members, respectively. Among the 73 HaPKs containing both PF07714 and PF00069 kinase domains, the RLK-Pelle_CrRLK1L-1 and RLK-Pelle_LRR-I-1 contain 27 and 25 members, respectively. HaPKs that retain several kinase domains during evolution likely work with specific substrates.

There are 1,038 members of the 2,583 HaPKs containing other conserved domains in addition to the kinase domain (**Supplementary Table S6**). The number of conserved domains they contain ranges from 1 to 10. There is only one HaPK with 10 domains including Zinc finger C3HC4 type (1), Ankyrin repeats (3), and Mind bomb SH3 repeat domain (6), and it belongs to the TKL-Pl-1 subfamily. Apart from the kinase domain, the most frequently occurring conserved domains in HaPKs are the Leucine-rich repeats domain, including Leucine-rich repeat 8 (LRR_8, PF13855), Leucine-rich repeat N-terminal domain 2 (LRRNT_2, PF08263) and Leucine Rich Repeat 1 (LRR_1, PF00560). In addition, other commonly occurring domains include D-mannose binding lectin (B_lectin, PF01453) and S-locus glycoprotein domain (S_locus_glycop, PF00954) (**Supplementary Table S8**). The diverse domains serve multiple functions in sunflower adaptation.

In order to explore the structural diversity of sunflower protein kinase genes, we examined the number of introns present in all kinase genes (**Supplementary Table S9**). The number of introns in HaPKs varies greatly, with the minimum being 0 and the maximum being 28. Most protein kinases contain very few introns. Statistical analysis revealed that 2,333 (90.3%) HaPKs have 10 or fewer introns, of which 591 HaPKs do not contain any introns. Only one HaPK contains 28 introns. To analyze the distribution trend of intron numbers among subfamily members, we calculated the variance for each subfamily (**Supplementary Table S10**). The results show that in most subfamilies, the number of introns is similar. In a few subfamilies, the degree of dispersion in intron numbers is relatively large. This analysis suggests that members of the same subfamily tend to have a certain similarity in their intron numbers (**Supplementary Figure 3A**). For example, 16 subfamilies have a variance of 0, indicating that their members have the same number of introns. The RLK-Pelle_WAK subfamily contains 107 members, with intron numbers ranging between 1 and 4. The RLK-Pelle_SD-2b subfamily contains 94 members, with most having 1 or 2 introns. Moreover, a comparison between gene structure and phylogeny revealed that members with a similar number of introns exhibited a closer relationship in the phylogenetic tree (**Supplementary Figures 3B, C**).

**Figure 3:**
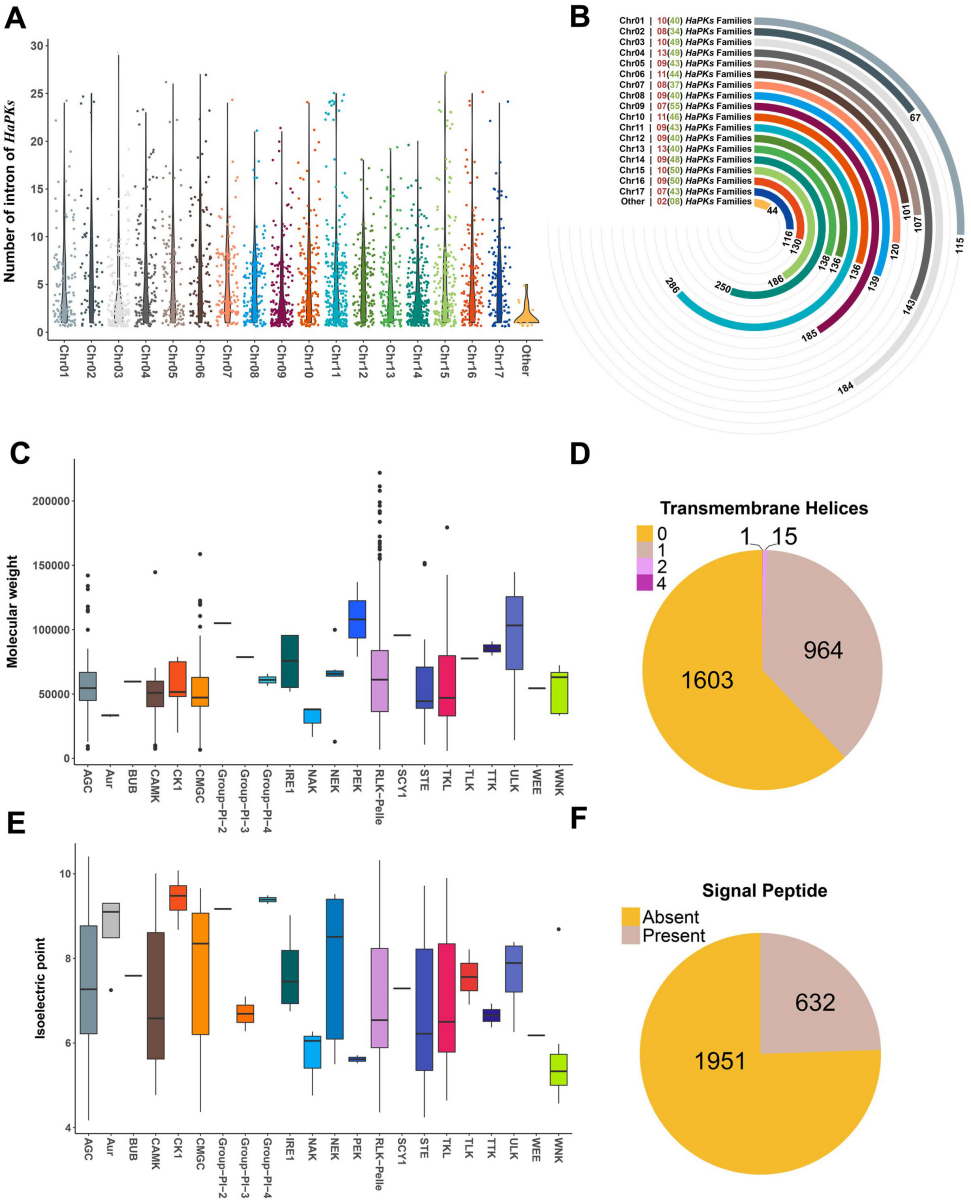
Descriptive analysis of characteristic of HaPKs. **(A)** Distribution of sunflower protein kinases on chromosomes and number of introns. Each point represents a protein kinase, with the Y-axis representing the number of introns in each protein kinase. **(B)** Count of sunflower protein kinase families and protein kinases on each chromosome. The numbers in brackets indicate the number of subfamilies, while the numbers outside brackets indicate the number of families. A bar chart represents the count of protein kinases on each chromosome. **(C, E)** Distribution of molecular weight and isoelectric point of various kinases in the protein kinase family. **(D, F)** Prediction of transmembrane domains and signal peptides of sunflower protein kinases.

In our analysis, we also examined the distribution of introns across each chromosome. We found that the quantity of introns was evenly distributed throughout all chromosomes, without any discernible concentration trend (**Figure 3A**). In terms of chromosome location, the majority of kinase genes were mapped to all 17 sunflower chromosomes, with only 44 genes located on the scaffold. The number of kinase genes per chromosome ranged from 67 to 286, with chromosome 11 and 14 having the largest number of genes, with 286 and 250 respectively (**Figure 3B**). At the family level, the number of families and subfamilies was comparable across each chromosome, with a range of 7 to 11 families and 34 to 55 subfamilies, respectively. Most subfamilies were distributed equally across several chromosomes, while certain subfamilies were concentrated on several chromosomes. Notably, the members of the RLK-Pelle_LRR-* subfamily exhibit a clear trend of uneven distribution on chromosomes. For example, in the three subfamilies RLK-Pelle_LRR-XI-1, RLK-Pelle_LRR-IX, and RLK-Pelle_LRR-I-1, the family members are primarily distributed on chromosomes 3, 14, and 11, respectively (**Supplementary Figure 4**).

We characterized the other protein properties for HaPKs including molecular weight, isoelectric point, transmembrane domain (TM) and signal peptide (SP) (**Figures 3C–F**). Most HaPKs did not contain TM or SP, and out of the PKs that contained TM (980), 97.77% (964) just had one transmembrane helix, fifteen contained two transmembrane helices and one contained four transmembrane helices (**Figure 3D**; **Supplementary Table S11**). Regarding to SP, there are 632 HaPKs possessing SP (**Figure 3F**; **Supplementary Table S12**). SPs are short peptides found at the N-terminal of proteins and are responsible for carrying essential information for protein secretion (Owji et al., 2018). Comprehensive analysis of HaPKs containing transmembrane domains or signal peptides revealed that all 632 HaPKs with signal peptides also contain transmembrane domains. By identifying their family classification, it was found that 630 of these HaPKs belong to the RLK-Pelle family. Further family classification of 980 HaPKs with transmembrane domains showed that 975 of them belong to the RLK-Pelle family. It is well known that the RLK-Pelle family is associated with extracellular signal perception, indicating that the presence of transmembrane domains and signal peptides in HaPKs is significantly related to their function in sensing extracellular signals.

### 3.3 The origin of the sunflower kinome: insights into gene duplication

**Figure 4:**
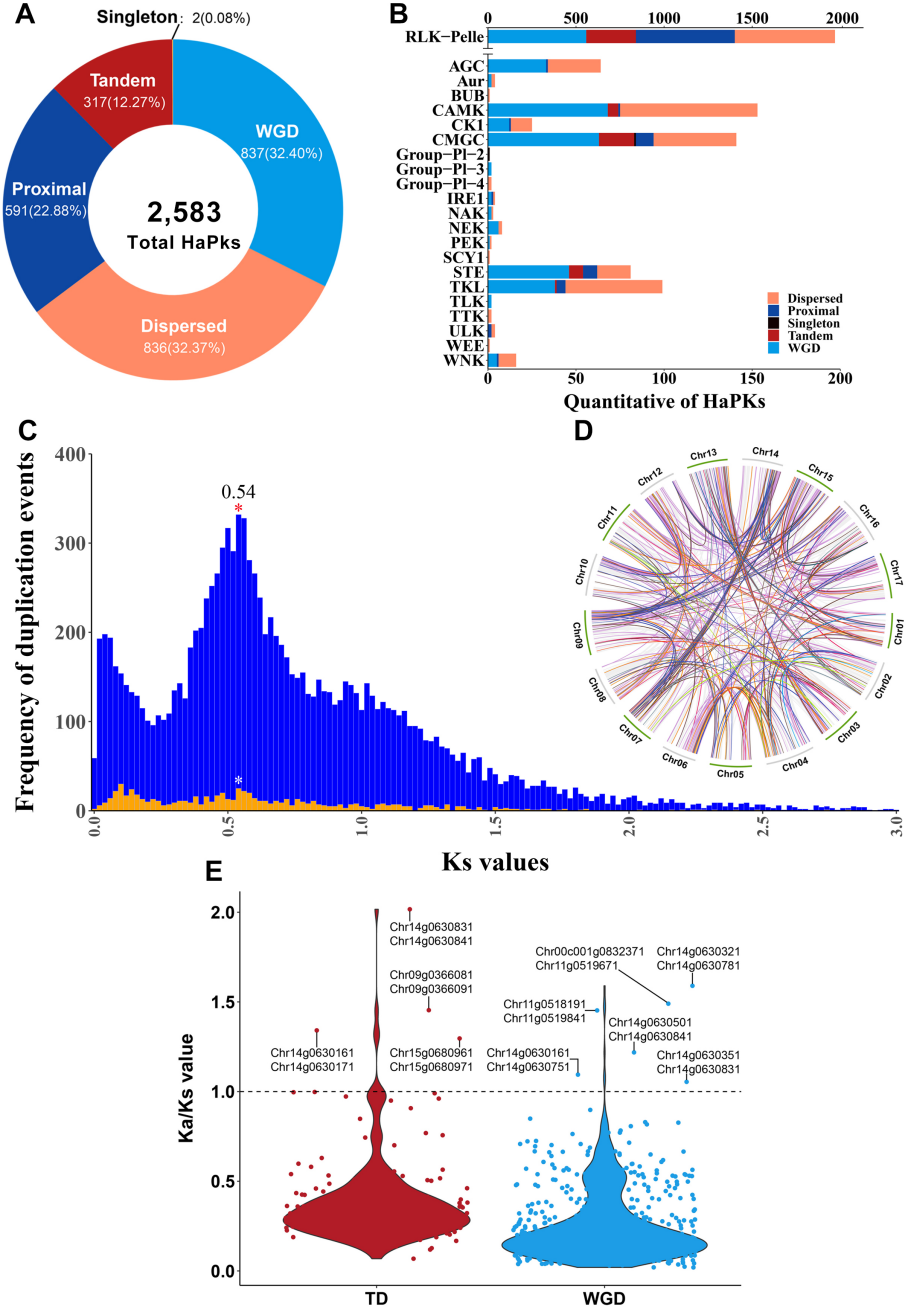
Gene duplication events. **(A)** Number of HaPKs by different gene duplication event. **(B)** The distribution pattern of different gene duplication event among the sunflower protein kinase families. **(C)** Ks values of PK collinearity. The background image displays the Ks values of all genes in the sunflower, while the foreground image represents the Ks values of HaPKs. **(D)** Collinear analysis of HaPKs. **(E)** The Ka/Ks values of collinear gene pairs. The text in the figure indicates gene pairs with a Ka/Ks value greater than 1.

The process of gene duplication plays a significant role in the evolution of plant genomes, offering a wealth of vital genetic resources that enable plants to develop new functions and adapt to constantly changing environments (Freeling, 2009). Gene duplication can be categorized into two main types: whole-genome duplication (WGD) and single-gene duplication (Qiao et al., 2018). Single-gene duplications can be further subdivided into tandem duplication (TD), proximal duplication (PD), and dispersed duplication (DSD) (Freeling, 2009). The 2,583 HaPKs were categorized into five distinct types: WGD (837, 32.40%), dispersed (836, 32.37%), proximal (591, 22.88%), tandem (317, 12.27%), and singleton (2, 0.08%) (**Figure 4A**; **Supplementary Table S13**). The expansion of HaPKs was primarily driven by two types of duplication events: WGD and DSD. WGD contributed to the expansion of 15 kinase families, while DSD played a significant role in the expansion of 19 kinase families (**Figure 4B**). Further analysis revealed that sunflower underwent a whole-genome duplication event of approximately 32.72 MYA (**Figure 4C**). This finding aligns with the results reported by Hélène Badouin (Badouin et al., 2017), which suggested that sunflower experienced WGD events around 29 MYA. After estimating collinear blocks, we identified a total of 569 gene pairs generated from WGD and 166 gene pairs generated from TD (**Figure 4D**; **Supplementary Table S14**). TD events, which occurred more rapidly than WGD, were crucial for plant environmental adaptation and were involved in the expansion of five kinase families, particularly the RLK-Pelle family, accounting for 88.96% of these events. The 166 collinear gene pairs were then mapped onto the sunflower genome, forming multiple tandem PK clusters across all 17 sunflower chromosomes (**Supplementary Figure 5**). Additionally, we observed a relatively balanced distribution of gene duplication events across the chromosomes, emphasizing the variations in the different types of duplication events (**Supplementary Figure 6**).

The ratio of non-synonymous to synonymous substitutions (Ka/Ks) was employed to assess the selection pressure on these homologous gene pairs. A Ka/Ks value greater than 1 indicates positive selection, a value less than 1 indicates negative selection (purifying selection), and a value of 1 suggests neutral evolution. Among the gene pairs from WGD, the Ka/Ks ratios ranged from 0.019 to 1.59, with a mean of 0.259. In the TD group, the ratios ranged from 0.068 to 2.016, with a mean of 0.392. Only six gene pairs from WGD and four gene pairs from TD had Ka/Ks values greater than 1, indicating that the majority of gene pairs were subject to purifying selection (**Figure 4E**).

### 3.4 Expression pattern of HaPKs in abiotic and biotic stresses

**Figure 5:**
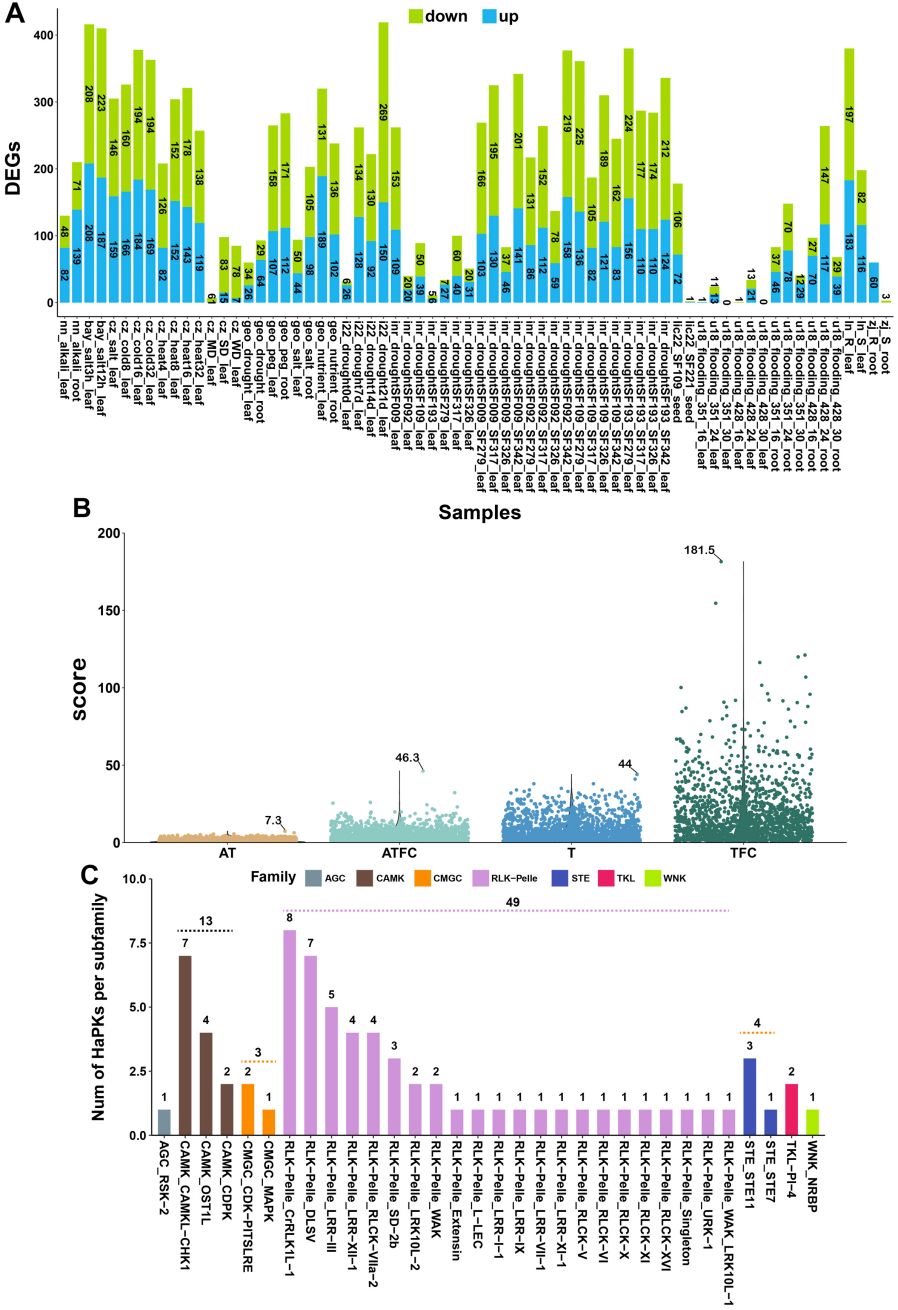
Transcriptome analysis of sunflower under different stress conditions. **(A)** Number of differentially expressed protein kinases. The X-axis represents the groups formed by comparing different treatment groups and control groups. The text provides information on the data source, type of stress, and corresponding sunflower tissues. **(B)** The scores obtained after scoring with four methods. The text in the figure indicates the maximum score obtained using each method. **(C)** Distribution of 73 significant protein kinases in the kinase family and subfamily. Different colors are used to represent different families, while the X-axis indicates subfamilies. The number on the dashed line represents the total number of kinases included in the family when it contains multiple subfamilies.

We conducted an analysis of the gene expression profiles of HaPKs using a comprehensive set of 10 publicly available datasets from the SRA database (**Table 1**). These datasets encompassed a wide range of stress conditions, including eight abiotic stresses (cold, heat, salt, alkali, drought, flooding, PEG6000, low-nutrient) and two biotic stresses (*Sclerotinia sclerotiorum* and *Orobanche cumana*) (**Table 1**). To identify PK genes differentially expressed under abiotic and biotic stresses, the PKs with |FC| > 2 (fold change) and p < 0.05 were retained (**Figure 5A**; **Supplementary Table S15**). Under different stress conditions, HaPKs were induced to be up-regulated or down-regulated in the majority of samples. For instance, in sunflower leaves subjected to drought stress for 21 days, we identified 150 up-regulated PKs and 269 down-regulated PKs. Similarly, in sunflower roots exposed to salt stress for 3 hours, we found 208 up-regulated PKs and 208 down-regulated PKs. Just in a few samples, either no PKs were expressed or only a limited number were detected. Based on these observations, we speculate that HaPKs play a significant role in sunflower’s stress response.

**Table 1:** Transcriptome data of sunflower under various biotic and abiotic stresses.

Source	Nation	Stress	Tissue	Num. of samp.	Project	Create date
NORTHEAST NORMAL UNIVERSITY	China	alkali	root leaf	12	SRP294448	2021-04-01
BAYANNAOER CITY INSTITUTE OF AGRICULTURE AND ANIMAL HUSBANDRY SCIENCE	China	salt	leaf	9	SRP391119	2022-08-11
CHANGZHI UNIVERSITY	China	cold heat drought salt	leaf	57	SRP392176	2022-08-15
UNIVERSITY OF GEORGIA	America	drought PEG6000 salt low-nutrient	root leaf	38	SRP326108	2021-06-30
INNER MONGOLIA AGRICULTURAL UNIVERSITY	China	drought	leaf	24	SRP355134	2022-01-17
INRAE	French	drought	leaf	142	SRP245841	2020-01-29
INRA-CNRS-Toulouse University	French	PEG6000	seed	12	SRP092742	2022-01-29
UNIVERSITY OF BRITISH COLUMBIA	Canada	flooding	root leaf	96	SRP162252	2018-12-27
LIAONING ACADEMY OF AGRICULTURAL SCIENCES	China	*Sclerotinia sclerotiorum*	leaf	12	SRP411503	2022-12-05
ZHEJIANG UNIVERSITY	China	*Orobanche cumana*	root	12	SRP411503	2018-12-27

Our objective was to identify the key PKs involved in sunflower responses to various stresses among the 2,583 PKs analyzed. Nevertheless, the presence of samples from various projects posed a challenge in fully eliminating the batch effect, making it impractical to employ conventional methods for data analysis. As a result, we opted to grade all the PKs using four different methods. Under various abiotic and biotic stress conditions, when a protein kinase shows up-regulation or down-regulation in the treatment group compared to the control group, it indicates that this protein kinase is involved in sunflower’s response to that specific type of stress. If a protein kinase is up-regulated, it suggests the kinase participates in the positive regulation of the stress response; if it is down-regulated, it suggests involvement in negative regulation. We believe that both positive and negative regulation of genes hold equal value in sunflower’s stress response, so they should be scored equally when evaluating protein kinases. We assessed the importance of protein kinases based on their frequency and intensity of response across all samples. If a protein kinase is up-regulated or down-regulated in a sample, it is scored as 1; otherwise, it is scored as 0. The total response frequency is the sum of all scores across samples. The intensity of response is measured by the fold change in differential expression; if a protein kinase is up-regulated or down-regulated, the fold change is used as the score; otherwise, it is scored as 0. The total response intensity is the sum of all fold-change scores across samples. Since the number of transcriptome samples varies across different stress conditions, calculating the total score alone would overemphasize genes in stress conditions with more samples. Therefore, we classified the samples by stress type and calculated the average score for each type of stress, then summed the averages to get the final score. Ultimately, we used four scoring methods to evaluate the importance of protein kinases in sunflower stress responses from different dimensions. Each method has its own emphasis, ensuring that important protein kinases are not overlooked. We named the four scoring methods sequentially as T (score by total), TFC (score by total of fold change), AT (score by average of total), and ATFC (score by average of total of fold change). Through analysis, we found that a total of 1,603 genes were up-regulated or down-regulated under different stress conditions. These genes were scored using four methods (T, AT, TFC, and ATFC), with the scoring ranges as follows: 1-44, 0.3-7.3, 1-181.5, and 0.04-46.3 (**Figure 5B**), respectively. Subsequently, we ranked the PKs based on the scores obtained from each method (**Supplementary Tables S16**–**S19**). Our goal was to identify PKs that displayed a broad spectrum of resistance to diverse stresses, thereby contributing to boosting sunflower productivity and sustainability. We obtained the top 30 PKs from each ranking and removed any duplicate PKs. In the end, we identified a total of 73 PKs (**Supplementary Table S20**). In exploring sunflower’s response to different stress conditions, they can serve as important candidate genes for further research.

**Figure 6:**
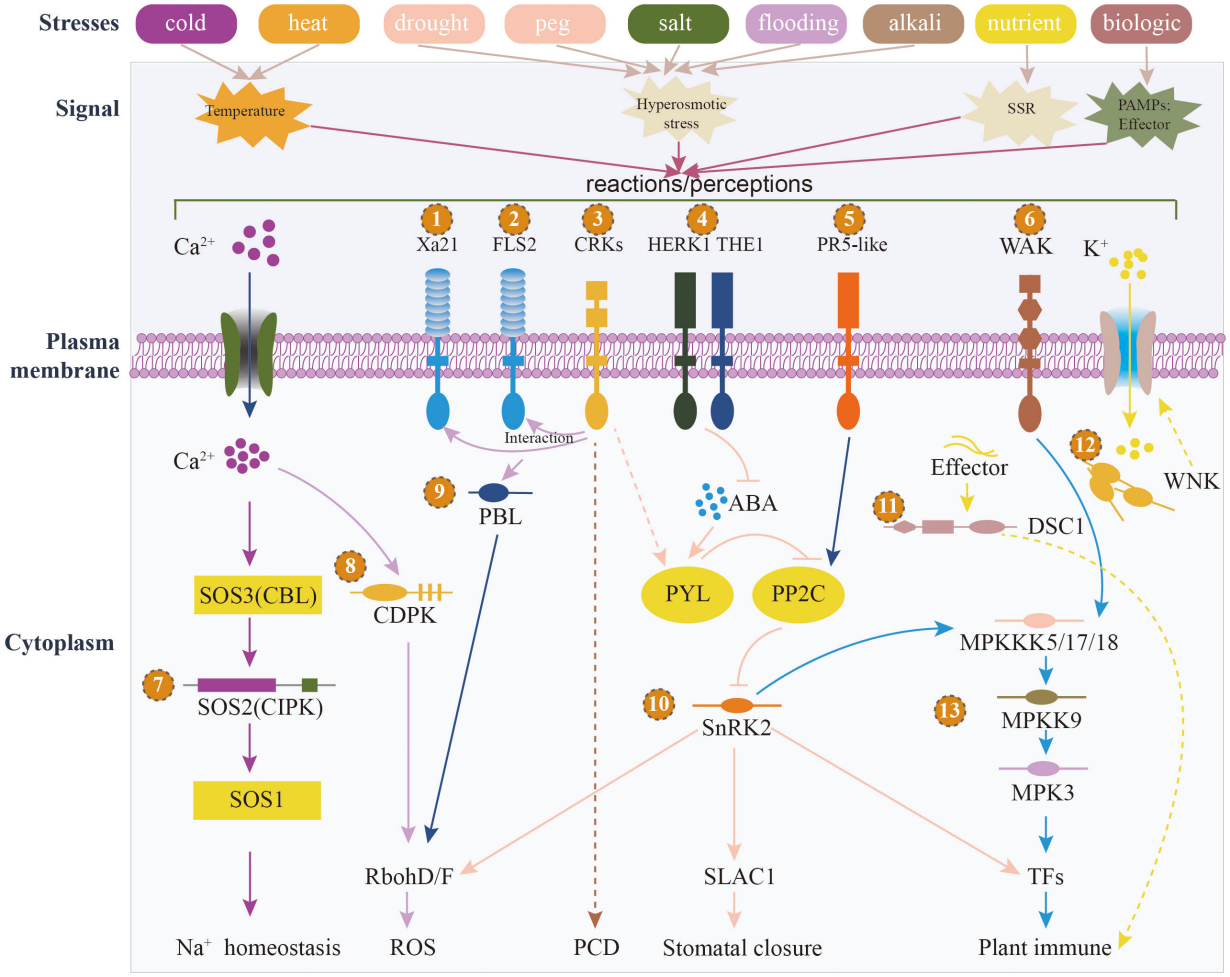
Functional postulated models of portion of 73 HaPKs are depicted in the figure. It illustrates the diverse types of abiotic and biological stresses, along with the corresponding signals they generate. The numbers 1-13 represent the significant genes annotated within the various signaling pathways.

The 73 identified PKs are distributed across 7 families and 32 subfamilies (**Figure 5C**; **Supplementary Table S20**). Among these, the RLK-Pelle and CAMK families have the highest number of members, with 49 and 13 members, respectively. In terms of subfamilies, RLK-Pelle_CrRLK1L-1 and CAMK_CAMKL-CHK1 contain the most members, with 8 and 7 members, respectively (**Figure 5C**). The findings revealed the significant involvement of the RLK-Pelle and CAMK families in the sunflower’s response to various stressors. Plants have an innate immune system that depends on cell surface and cytoplasmic immune receptors to detect and respond to stress signals (Dodds and Rathjen, 2010). These receptors, known as pattern recognition receptors (PRRs) located on the cell membrane, and intracellular immune receptors called nucleotide-binding domain leucine-rich repeat containing proteins (NLRs), act as sensors to monitor molecular patterns released by microbes or plants, as well as the effectors that pathogens specifically release into plant cells (Tang et al., 2017). When these receptors detect these patterns or effectors, they initiate signal transmission, leading to transient calcium influx, production of reactive oxygen species (ROS), activation of MAPKs, and ultimately triggering a range of defense mechanisms that restrict the progression of pathogens (Qi et al., 2017). To gain insights into the roles and mechanisms of these 73 PKs in sunflower’s response to different stresses, we employed a range of databases, including SMART, Pfam, STRING, TAIR, and the NCBI web blastp tool (**Supplementary Table S21**). Through the utilization of these resources, we annotated the functions of these PKs, revealing their involvement in significant pathways such as MAPK signaling, plant hormone signal transduction, plant-pathogen interaction, and autophagy (**Figure 6**). We explored several PKs belonging to PRR, such as HanXRQr2_Chr08g0352561 and HanXRQr2_Chr13g0573541. These two PKs play a crucial role in sensing the integrity of cell walls and can be activated by various stressors, including wounding, pathogen infection and other abiotic stress conditions (Gigli-Bisceglia et al., 2022).

Additionally, we identified several RLCKs, which are signaling proteins that regulate plant cellular activities in response to both biotic and abiotic stresses. For instance, the PK HanXRQr2_Chr06g0264671 is a PBL protein belonging to the RLCK family. It can phosphorylate the nicotinamide adenine dinucleotide phosphate (NADPH) oxidase RESPIRATORY BURST OXIDASE HOMOLOG D (RBOHD), leading to the induction of apoplastic ROS production (Chu et al., 2023).

Our research uncovered a significant presence of CIPKs, which exhibited up-regulation in response to various stressors encountered by sunflowers. In the Salt Overly Sensitive (SOS) pathway, an EF-hand Ca^2+^ binding protein known as SOS3/CBL interacts with and activates SOS2, a protein belonging to the CIPKs family (also known as SnRK3). CIPKs represent a subclass of serine/threonine protein kinases that interact with CBL, a Ca^2+^ sensor, governing the regulation of growth, development, and stress responses in plants. The interaction between CIPKs and CBL plays important roles in Ca^2+^-mediated responses to various stresses, including the ability to respond to environmental stresses like salt, cold, and drought, as well as in managing biotic stress, particularly in regulating ion transporter activities (Yang et al., 2022). For instance, HanXRQr2_Chr09g0371011, a member of the CIPKs family, demonstrated up-regulation under cold, heat, salt, and drought conditions.

Through our investigation, we found that multiple PKs were members of the SnRK2 family which play a crucial role in the ABA-activated signaling pathway. SnRK2s serve as a vital mediator for various plant responses, including stomatal closure, ROS production, transcription of cold-responsive genes, and adaptation to hyperosmotic stress induced by salt, drought (Zhang et al., 2022). Remarkably, regardless of the methods employed, we consistently found that the PK HanXRQr2_Chr13g0607501 always was first in line. Belonging to the SnRK2 family, its presence further underscores the significant role played by SnRK2s in sunflowers’ adaptation to diverse environmental conditions and in combating pathogen infections. The expression levels of these 73 PKs were observed to be down-regulated or up-regulated in sunflowers in response to diverse stresses, signifying their potential contribution to the growth and development of sunflowers under varying environmental conditions. The underlying mechanisms involved in this process warrant further investigation.

### 3.5 Expression profiles of HaPKs in specific tissue and exposure to various phytohormone

**Figure 7:**
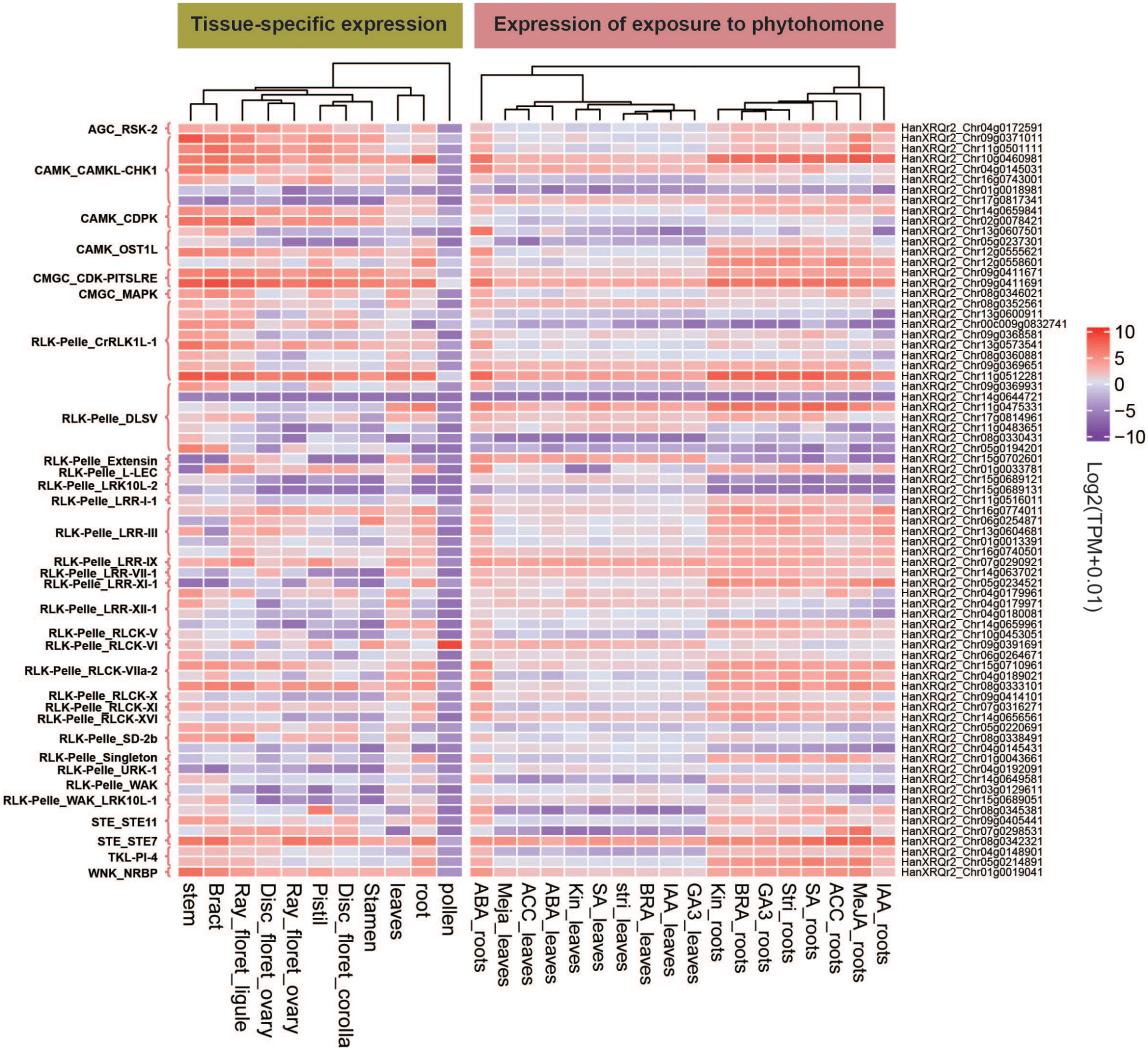
Transcriptome data of tissue-specific expression and hormone-induced expression of 73 HaPKs. The horizontal coordinate represents 11 sunflower tissues and 9 hormones, while the vertical coordinates display the names and subfamilies of the 73 genes.

We analyzed the expression data of all 2,583 HaPKs in 11 different tissues of sunflower, including root, stem, leaf, bract, pollen, stamen, pistil, DF ovary, DF corolla, RF ovary, and RF ligule, by screening TPM (transcripts per million) values. Specifically, we focused on the expression profiles of 73 PKs obtained earlier and visualized them using a heatmap (**Figure 7**; **Supplementary Table S22**). Using a Tau value calculation, we established a threshold of 0.8 to identify tissue-specific HaPKs. This led to the identification of 44 tissue-specific HaPKs, with the majority concentrated in the roots, stems, and leaves. Through our clustering analysis of the expression profiles, we discovered that the 11 tissues could be classified into five distinct groups: (1) root and leaf; (2) stamen, DF corolla, and pistil; (3) DF ovary, RF ovary, and RF ligule; (4) bract and stem; and (5) pollen. Notably, most PKs showed low expression levels in pollen, except for HanXRQr2_Chr09g0391691, which exhibited highly specific expression in pollen and relatively low expression in other tissues. This observation suggests that HanXRQr2_Chr09g0391691 likely plays a crucial role in pollen-related processes. Besides being specifically expressed in pollen, numerous other genes exhibit tissue-specific expression patterns. For instance, HanXRQr2_Chr08g0345381 is specifically expressed in the pistil, while HanXRQr2_Chr05g0234521, HanXRQr2_Chr11g0475331, and HanXRQr2_Chr12g0558601 are specifically expressed in roots. HanXRQr2_Chr04g0192091 shows specific expression in leaves, and HanXRQr2_Chr05g0194201 and HanXRQr2_Chr09g0371011 are specifically expressed in stems. Moreover, certain PKs display high expression levels in all tissues except pollen. Notable examples include HanXRQr2_Chr09g0411691 and HanXRQr2_Chr09g0411671, which are involved in regulating the cell cycle, along with the MAPK pathway gene HanXRQr2_Chr08g0342321. On the other hand, there are genes that show little to no expression in most or all tissues, such as HanXRQr2_Chr14g0644721 in the RLK-Pelle_DLSV subfamily, as well as HanXRQr2_Chr01g0018981 and HanXRQr2_Chr17g0817341 in the CAMK_CAMKL-CHK1 subfamily.

Besides analyzing tissue-specific expression data, we also extracted the expression profiles of HaPKs in roots and leaves after exposure to various plant hormones. We selected 73 significant PKs from the entire set of HaPKs and visualized their expression profiles using a heatmap (**Figure 7**; **Supplementary Table S23**). Based on the clustering analysis of expression profiles, the data were categorized into three groups: ABA-treated root, other-treated roots and hormone-treated leaves. Notably, the expression data of ABA-treated roots formed a distinct category, providing evidence for the crucial role of ABA signaling pathways in a plant’s response to diverse stresses. The results also demonstrate that within the same branch, the HaPKs exhibit similar expression patterns under different hormone treatments. Upon comparing the tissue-specific expression data with the plant hormone induction data separately in roots and leaves, we observed that the expression patterns of the majority of PKs were similar, suggesting that several PKs were not induced upon hormone treatment. For instance, in the tissue-specific expression data, the expression of HanXRQr2_Chr10g0460981 in roots was 226.83, whereas the highest expression observed under hormone induction in roots was 166.33. Similarly, the tissue-specific expression data showed that the expression level of HanXRQr2_Chr09g0411691 in roots was 107.99, while the highest expression level observed under hormone induction in roots was 128.84. Nevertheless, hormone induction led to a significant increase in the expression of certain PKs. For instance, under ABA induction, the expression levels of HanXRQr2_Chr13g0607501 and HanXRQr2_Chr04g0145031 in roots increased by 3084-fold and 20-fold, respectively. Similarly, under MeJA induction, the expression levels of HanXRQr2_Chr11g0501111, HanXRQr2_Chr07g0298531, and HanXRQr2_Chr07g0298531 in roots were amplified by 21-fold, 22-fold, and 91-fold, respectively.

### 3.6 The analysis of domain of HaPKs

**Figure 8:**
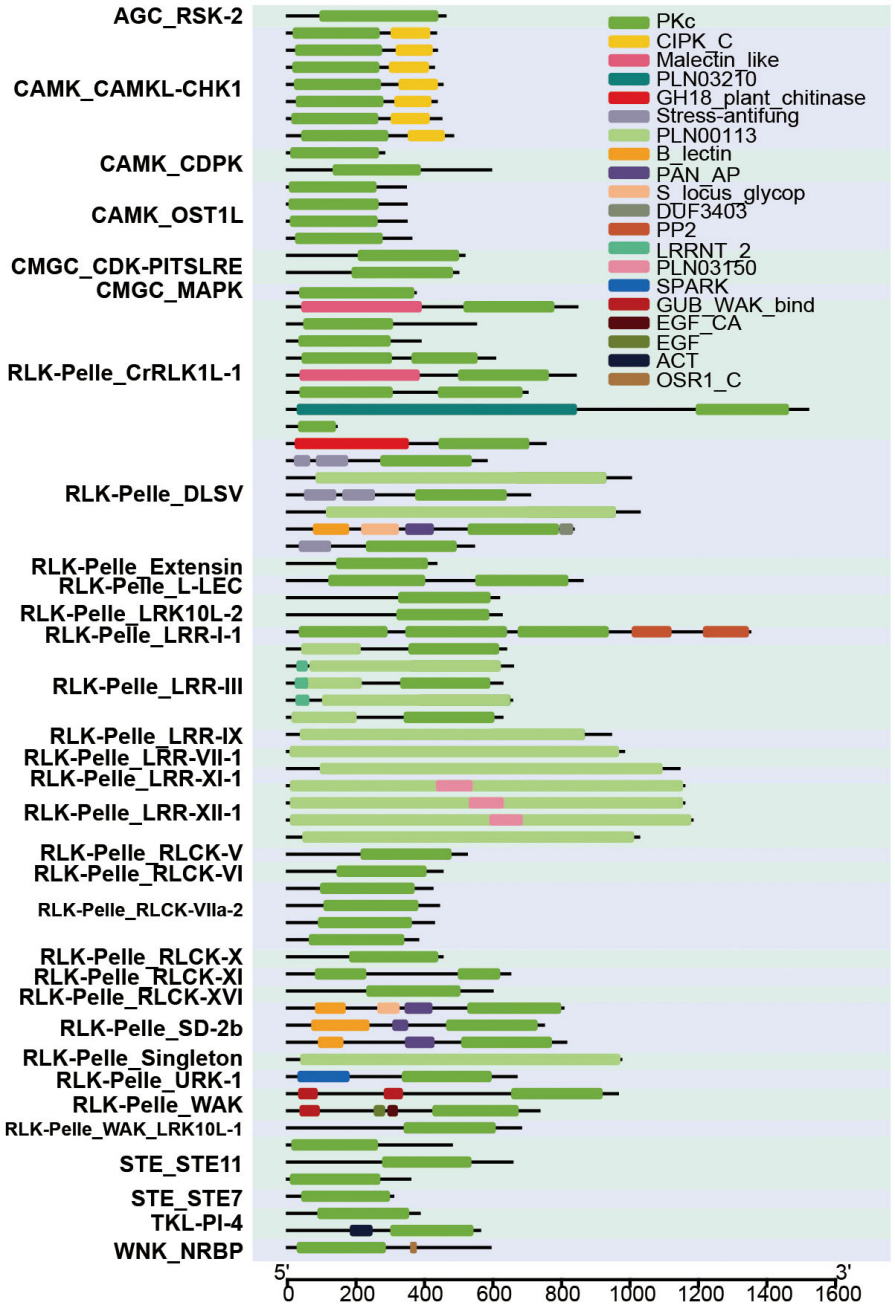
Prediction of domain of 73 HaPKs. The horizontal coordinate represents the number of amino acids present in each protein. The ordinates display the names and subfamilies of the 73 protein kinases. Different colors in the figure indicate the presence of different domains.

We acquired domains of 2,583 HaPKs and visually represented the findings for 73 PKs (**Figure 8**). Among these, a total of 20 domains were identified; certain domains were conserved across all PKs, while others were specific to certain PKs. For example, the PKc domain was found to be highly conserved in almost all 73 PKs. However, some PKs lacked the PKc domain due to its integration into the PLN00113 domain, resulting in these PKs exhibiting only the PLN00113 domain, as seen in a subset of the RLK-Pelle-LRR members. Certain domains are exclusive to specific subfamilies, such as the CIPK_C domain, which is found solely in the CAMK_CAMKL-CHK1 subfamily. This subfamily is characterized by a C-terminal regulatory domain associated with Ca^2+^ and represents a distinct protein family in higher plants. These proteins interact with calcineurin B-like (CBL) calcium sensors, forming a signaling network that interprets specific calcium signals induced by various environmental stimuli, including salinity, drought, cold, light, and mechanical perturbations, among others. The RLK-Pelle_DLSV subfamily stands out for its exclusive possession of the Stress-antifung domain, which showcases six conserved cysteines that play a crucial role in forming disulphide bridges. Functionally, this domain is involved in responding to salt stress and exhibits antifungal activity.

## 4 Discussion

Using the HMMER v3.2.1, phylogenetic analysis, and classification by iTak software, a total of 2,583 HaPKs were identified, comprising 3.62% (71,289) of the total number of putative proteins in sunflower. This percentage aligns with protein kinase studies conducted in other species, such as 3.25% in common bean, 3.7% in grapevine, and 4.7% in soybean. The HaPK family is one of the largest in sunflower, and the abundance of protein kinases can be attributed, in part, to the plant’s large genome size and a whole-genome duplication (WGD) event that took place approximately 32.72 million years ago (Mya). This highlights the significance of protein kinases in sunflower growth, development, and ability to adapt to diverse stress conditions. We divided the 2,583 HaPKs into 22 families and 121 subfamilies, which is also similar to the numbers in soybean (122), *Arabidopsis* (119), and grapevine (121). Among all families, the RLK-Pelle family was the largest, consisting of 1,967 members and accounting for 76.2% of all identified kinases. The RLK-Pelle family exhibited a significantly higher expansion rate compared to other families, mainly due to the expansion of multiple subfamilies through tandem duplication. This rapid expansion likely results from their adaptation to fast-evolving pathogens (Lehti-Shiu et al., 2009). The RLK-Pelle family derives its name from the presence of receptor-like kinases (RLKs) structure and its close resemblance to the Pelle family of kinases found in animals (Shiu and Bleecker, 2001). Interestingly, the genes encoding plant RLK-Pelle proteins exhibit closer evolutionary relationships with kinase-encoding genes in animals than with other plant protein kinases from classes such as CMGC-MAPKs and CAMK-CDPKs. Except for the RLK-Pelle family, certain PK families in sunflower exhibited a limited number of members, suggesting that their roles may be more focused on fundamental cellular processes rather than stress response mechanisms (Shiu et al., 2004).

Within the RLK-Pelle family, it is further classified into 57 subfamilies (**Figure 1B**). Among these subfamilies, DLSV and CrRLK1L were the largest, boasting 361 and 335 members respectively, surpassing all other subfamilies by a significant margin. Notably, the DLSV family members accounted for 13.98% of the total protein kinases identified in sunflower, a proportion that aligns with other species such as cowpea (13.46%) and grapevine (13.53%). However, the CrRLK1L family expanded significantly in sunflower, accounting for 12.97% of the total protein kinases (**Figure 2**; **Supplementary Table S5**), which is much larger than the proportion of CrLK1L family in other species, such as 3.25% in cowpea, 2.65% in grapevine, and 2.86% in soybean. CrRLK1L family is unique to plants, and homologous genes have not been found in animals and microorganisms; they were first identified in *Catharanthus roseus* (Schulze-Muth et al., 1996). It is located on the cell membrane and contains a special extracellular domain. Its main functions include information exchange between male and female gametophytes during sexual reproduction, perception of cell wall integrity during vegetative growth, and regulation of cell elongation. More representative members of this family include ANX, FER, THE1, HERK, which are involved in the processes of plant fertilization (Escobar-Restrepo et al., 2007; Miyazaki et al., 2009), as well as sensing cell wall cellulose integrity (Guo et al., 2009a; Guo et al., 2009b) playing an important role. Interestingly, similar to the CrRLK1L family, the WAK family has a considerably larger number of members in sunflowers than in any other species. In sunflower, the WAK family comprises 107 members, representing 4.14% of the total protein kinases. However, the presence of WAK family members is significantly lower in other species. For instance, there are only 22 members (1.02%) in soybean, 14 members (1.16%) in common bean, and 30 members (2.57%) in grapevine. WAK, short for Wall-associated kinase, is a group of receptor-like proteins that possess a cytoplasmic serine/threonine kinase, a transmembrane domain, and a less conserved region that is anchored to the cell wall, containing a series of epidermal growth factor repeats. Emerging evidence suggests that WAKs function as receptors for both short oligogalacturonic acid fragments generated during pathogen exposure or wounding, as well as longer pectins present in native cell walls. The WAK family plays a crucial role in various aspects of plant growth and development, as well as in defense against pathogens. In *Arabidopsis thaliana*, AtWAK1 and AtWAK2 have been found to be involved in cell wall elongation (Wagner and Kohorn, 2001). Additionally, quantitative resistance to *Fusarium* (Diener and Ausubel, 2005), *Verticilium* (Häffner et al., 2014), *Sporisorium reilianum* (Zuo et al., 2015), and *Exserohilum turcicum* (Hurni et al., 2015) has been observed in both *Arabidopsis* and maize, mediated by WAK family members. Both the CrRLK1L and WAK families, which are associated with plant cell wall development, have undergone significant expansion in sunflowers. It is speculated that these expanded families may contribute to the stress resistance of sunflowers, enhancing the mechanical strength of cell walls, thereby equipping them to adapt to their environment and effectively prevent pathogen invasion.

The conservation of intron/exon structures in PKs, which are linked to growth and development processes, may have originated with the emergence of land plants and subsequently persisted (Yan et al., 2017). This conservation is frequently utilized as an indicator of genetic diversity. In terms of intron distribution in HaPKs, the mean number of introns (4.14) is lower than that of other species, such as strawberry (6.45) (Liu et al., 2020) and common bean (5.74) (Aono et al., 2023). The highest intron number observed was 28 in the PEK_GCN2 subfamily, which is the same number found in common bean (Aono et al., 2023) and cowpea (Ferreira-Neto et al., 2021). Furthermore, 591 HaPK genes (22.9%) did not possess introns, which is significantly higher than other species, such as wheat (11.9%) (Wei and Li, 2019) and soybean (12.1%) (Liu et al., 2015). Notably, the RLK-Pelle_CrRLK1L-1 subfamily had 153 (45.67%) members that did not contain introns. At the family level, there is compelling evidence indicating a correlation between the evolutionary trajectory of gene families and the structural diversity exhibited by their constituent genes. The gene structures of HaPK subfamilies revealed distinct tendencies towards either intron loss or acquisition. The variance analysis of the number of introns contained by members within subfamilies shows that the values range from 0 to 112 (**Supplementary Table S10**). This indicates that while some subfamilies have members with similar intron counts, others show considerable differences among members. This suggests that even within the same subfamily, there is no consistent pattern in the number of introns among its members.

In our study, we conducted an analysis to determine the number of kinase domains present in each HaPK. The results showed a range of 1 to 6 kinase domains. Interestingly, we identified 189 PKs (7.3% of the total) that contained more than one kinase domain, and which were distributed across 25 subfamilies. The distribution of kinase domains observed in HaPKs closely resembled that found in sorghum (Aono et al., 2021) and grapevine (Zhu et al., 2018b). However, the situation was quite different in strawberry (Liu et al., 2020) and cowpea (Ferreira-Neto et al., 2021), where a strikingly high percentage of PKs (96.4% and 99.5%, respectively) contained more than two kinase domains. Regarding the distribution of kinase domains within subfamilies, the AGC_RSK-2 subfamily stood out, with 31 members (75.6% of the subfamily) containing 2 kinase domains, making it the subfamily with the highest proportion of members possessing multiple kinase domains. This finding aligns with previous studies conducted on common bean (Aono et al., 2023), sugarcane (Aono et al., 2021), and pineapple (Zhu et al., 2018a). In addition to AGC_RSK-2, the CMGC_SRPK and RLK-Pelle_LRR-I-1 subfamilies also exhibited multiple kinase domains, with 6 members (75%) and 37 members (36.3%), respectively. Notably, the RLK-Pelle_CrRLK1L-1 subfamily had the highest number of members (67) with multiple kinase domains, although this accounted for only 20% of the total subfamily members. The presence of multiple kinase domains in HaPKs may correspond to specific substrates, and further investigation into these kinases could provide valuable insights into the regulatory mechanisms governing kinase-substrate interactions.

The sunflower kinome exhibited a significant proportion of PK gene pairs with a Ka/Ks ratio below 1, suggesting that they have undergone purifying selection. This indicates that selection has played a role in preserving the structure and maintaining the functionality of PKs throughout their evolutionary history. In eukaryotes, this phenomenon is believed to occur during an initial phase of relaxed constraint or near-neutrality, which promotes diversification (Lynch and Conery, 2000). Similar to other biological processes, PK evolution may have experienced this phase due to their crucial significance in diverse biological processes (Janitza et al., 2012). We identified a distinct peak in Ks values around 0.54, indicating that the sunflower underwent a whole-genome duplication event approximately 32.75 MYA. This timing closely aligns with a major whole-genome duplication event estimated to have occurred 29 MYA in sunflower (Badouin et al., 2017). Our analysis revealed that this whole-genome duplication mechanism played a significant role in the expansion of the HaPK family, contributing to approximately 32.4% (837) of HaPKs. Notably, specific PK subfamilies, particularly those belonging to the RLK-Pelle group (RLK-Pelle_DLSV, RLK-Pelle_CrRLK1L-1, RLK-Pelle_SD-2b, RLK-Pelle_WAK, and RLK-Pelle_WAK), exhibited a more pronounced occurrence of tandem duplications. Tandemly duplicated PKs are known to be associated with stress responses (Qiao et al., 2018), suggesting that the expansion of these subfamilies has likely broadened their functional scope.

Protein kinases play a crucial role in various aspects of plant growth and development, as well as in responding to biotic and abiotic stresses. They are involved in important processes such as plant meristem development, leaf morphogenesis, reproductive growth, and yield (Zhu et al., 2023). For example, in *Arabidopsis thaliana*, members of the RLK-Pelle family, such as CLV1 and ERECTA, have been identified as regulators of plant growth and development (Cui et al., 2018). Furthermore, numerous studies have highlighted the significance of protein kinases in stress responses. One example is the WAK protein, a cell wall-associated protein kinase that senses the integrity of the cell wall by binding to pectin. In rice, it has been observed that OsWAK14, OsWAK91, and OsWAK92 contribute to enhancing resistance against rice blast, a common fungal disease (Li et al., 2019). Two protein kinases, TaXa21 and TaCRK10, have been successfully isolated from wheat. These kinases have the ability to interact with the transcription factor WRKY, thereby enhancing wheat’s resistance to stripe rust, particularly under high-temperature conditions (Costa et al., 2016). In addition to their role in improving plant resistance against biotic stress, protein kinases also play a crucial role in coping with abiotic stress. The ABA pathway plays a vital role in regulating osmotic pressure during drought and salt stress. Within this pathway, SnRK2 serves as a key regulator, orchestrating processes such as stomatal closure and the production of ROS (Trinh et al., 2014; Collin et al., 2021).

Duriez successfully cloned a protein kinase of the RLK-Pelle family, HAOR7, from sunflowers to enhance their resistance to broomrape (Duriez et al., 2019). However, there are few studies on protein kinases in sunflower. To gain a comprehensive understanding of the role of protein kinases in sunflower growth, development, and stress response, we collected multiple transcriptome datasets from the SRA. These datasets enabled us to observe the expression profiles of HaPKs under different conditions and assess their roles in sunflower growth, development, and stress response. The transcriptome data can be categorized into three parts: (1) HaPKs expression data under various biotic and abiotic stresses, including eight abiotic stresses (cold, heat, salt, alkali, drought, flooding, PEG6000, low-nutrient) and two biotic stresses (*Sclerotinia sclerotiorum* and *Orobanche cumana*); (2) HaPKs expression data induced by various plant hormones, including ABA, SA, MeJA, IAA, BRAS, ACC, GA3, and kinetin; and (3) tissue-specific expression data of HaPKs in different parts of sunflower, including root, stem, leaf, bract, pollen, stamen, pistil, DF ovary, DF corolla, RF ovary, and RF ligule.

We conducted a transcriptomic analysis on the raw data obtained from sunflowers subjected to various stresses. Through this analysis, we identified genes that exhibited up-regulated or down-regulated expression under each specific stress condition. Remarkably, we observed that HaPKs were consistently modulated in response to almost all types of stress (**Figure 5A**), highlighting their crucial involvement in plant stress responses, both biotic and abiotic in nature. Within our analysis, a total of 1,603 HaPKs were found to participate in these stress response processes. Given the substantial number of HaPKs identified, our focus shifted towards identifying those that confer broad-spectrum resistance in sunflowers. Such protein kinases hold significant potential for enhancing sunflower resistance breeding efforts and ultimately increasing crop yield. We considered the number of up-regulated and down-regulated HaPKs, as well as the fold change in their expression levels, as crucial indicators for assessing the significance of these kinases across all stress samples collected. Based on these indicators, we identified 73 HaPKs of considerable importance. Subsequently, we performed functional annotations on these 73 protein kinases and discovered their involvement in several vital stress regulatory processes in sunflowers. Protein kinases, including HanXRQr2_Chr09g0371011 and HanXRQr2_Chr11g0501111 from the CIPK family, play a crucial role in regulating Na^+^ homeostasis under salt stress by interacting with CBL proteins in response to Ca^2+^ influx signals. Meanwhile, HanXRQr2_Chr13g0607501 and HanXRQr2_Chr05g0237301 from the SnRK2 family serve as key regulators in the ABA signaling pathway. These kinases are involved in regulating sunflower resistance to drought and salt stress by controlling stomatal closure and ROS production. Furthermore, we have identified multiple pattern recognition receptors, such as those from the THE1, HERK1, PR5-like, WAK, and Xa21 families, located on the cell membrane. These RLK-Pelle family kinases can sense extracellular signals and transmit them through intracellular kinase domains. In addition to the aforementioned kinases, we have also discovered numerous other important kinases that contribute to our understanding of the resistance mechanisms employed by sunflowers.

Housekeeping genes, such as proteasome and ribosomal genes, maintain the fundamental structure and function of cells and are stably expressed across all cell types. In contrast, tissue-specific expression genes are only expressed in one or a few tissues, indicating that their function is specific to those tissues. Evaluating the tissue specificity of a gene is a crucial step in understanding its function (Dezső et al., 2008). We gathered TPM expression data for HaPKs across 11 plant tissues and conducted an in-depth analysis of 73 significant HaPKs. We identified 44 tissue-specific expression HaPKs, with the majority concentrated in the roots, stems, and leaves. The specific functions of each protein kinase in a given tissue require further investigation. Plant hormones play a crucial role in plant growth, development, and their ability to withstand environmental stress. Numerous studies have highlighted the significance of plant hormones in various plant processes. For instance, the application of exogenous jasmonic acid has been shown to enhance plant tolerance to cold (Cao et al., 2009), drought (Mohamed and Latif, 2017), salt (Shahzad et al., 2015), and heavy metals (Ahmad et al., 2017). In *Vigna angularis*, the exogenous application of salicylic acid regulates plant growth, development, photosynthesis, and mitigates damage caused by salt stress (Ahanger et al., 2019). Similarly, under salt stress, the application of brassinolide in tomatoes induces the accumulation of ethylene and hydrogen peroxide, leading to increased antioxidase activity (Zhu et al., 2016). To investigate the response of HaPKs to exogenous plant hormones, we obtained data on sunflowers treated with eight different plant hormones and analyzed the expression profiles of 73 key genes in both the roots and leaves. Following ABA treatment, HaPKs were found to be induced in the roots, indicating their active response to ABA signals. This finding aligns with our previous analysis, which suggested that the ABA signaling pathway plays a crucial role in sunflower stress resistance. Additionally, MeJA, ethylene, and auxin induced varying degrees of HaPK expression in sunflower roots. However, only a few PKs were induced in sunflower leaves, potentially due to the direct hormone treatment of the plant roots during the experiment.

## 5 Conclusion

Sunflower, a globally cultivated oil crop, holds significant importance due to its applications as cooking oil and animal feed in the form of sunflower cake. However, the escalating challenges posed by environmental changes, such as climate warming and increasing pollution, impose various risks on sunflower cultivation. In this context, protein kinases emerge as crucial players in the plant’s response to both biotic and abiotic stresses. Our investigation identified 2,583 HaPKs in sunflower, which were further categorized into 22 families and 121 subfamilies. We delved into the chromosome distribution, gene structural diversity, and evolutionary processes of these HaPKs, both at the family and gene levels, which enabled us to gain a preliminary understanding of the protein kinase landscape in sunflower. Focusing on the role of protein kinases in sunflower stress responses, we collected transcriptome data from sunflower plants worldwide, ensuring the exclusion of low-quality data. Consequently, we obtained sunflower expression data related to eight abiotic stresses (cold, heat, salt, alkali, drought, flooding, PEG6000, low-nutrient), two biotic stresses (*Sclerotinia sclerotiorum*, *Orobanche cumana*), eleven tissues (root, stem, leaf, bract, pollen, stamen, pistil, DF ovary, DF corolla, RF ovary, RF ligule), and eight plant hormones (ABA, SA, MeJA, IAA, BRAS, ACC, GA3, and kinetin). Through a comprehensive analysis of these transcriptome datasets, we successfully screened and annotated 73 crucial HaPKs, which predominantly participate in signaling pathways such as MAPK, plant hormone response, plant-pathogen interaction, and autophagy. Our study has provided initial insights into the role of these kinases in cell signal transduction processes, offering a new perspective for investigating protein kinases in sunflower. Furthermore, this work establishes a foundation for future research on the functional characterization of protein kinases in sunflower’s response to stress, growth, and development, employing genetic and molecular biology approaches.

## Data Availability

The original contributions presented in the study are included in the article/**Supplementary Material.** Further inquiries can be directed to the corresponding authors.

## Supplementary material

Supplementary Figure 1 Scoring method of sunflower protein kinase under different biological and abiotic stresses. FC, Fold Change.

Supplementary Figure 2 Analysis of sunflower protein kinase phylogenetic tree.

Supplementary Figure 3 The distribution trend of protein kinase introns within subfamilies and its relationship with genetic distances in the phylogenetic tree. **(A)** Distribution trend of protein kinases introns within subfamilies. **(B, C)** The relationship between the number of introns in protein kinases and the branches they occupy in the phylogenetic tree.

Supplementary Figure 4 Chromosomal distribution of sunflower protein kinase family.

Supplementary Figure 5 Chromosomal locations of the 166 tandemly arrayed sunflower PK gene pairs. This figure uses different colors to indicate different gene families.

Supplementary Figure 6 The distribution trend of protein kinase duplication events on chromosomes.

## References

[B1] Ahanger M. A. Aziz U. Alsahli A. A. Alyemeni M. N. Ahmad P. (2019). Influence of exogenous salicylic acid and nitric oxide on growth, photosynthesis, and ascorbate-glutathione cycle in salt stressed Vigna angularis. Biomolecules 10, 42. doi: 10.3390/biom10010042 31888108 PMC7022326

[B2] Ahmad P. AlYemeni M. N. Wijaya L. Alam P. Ahanger M. A. Alamri S. A. (2017). Jasmonic acid alleviates negative impacts of cadmium stress by modifying osmolytes and antioxidants in faba bean (Vicia faba L.). Arch. Agron. Soil Sci. 63, 1889–1899. doi: 10.1080/03650340.2017.1313406

[B3] Almagro Armenteros J. J. Tsirigos K. D. Sønderby C. K. Petersen T. N. Winther O. Brunak S.. (2019). SignalP 5.0 improves signal peptide predictions using deep neural networks. Nat. Biotechnol. 37, 420–423. doi: 10.1038/s41587-019-0036-z 30778233

[B4] Aono A. H. Pimenta R. J. G. Da Silva Dambroz C. M. Costa F. C. L. Kuroshu R. M. De Souza A. P.. (2023). Genome-wide characterization of the common bean kinome: Catalog and insights into expression patterns and genetic organization. Gene 855, 147127. doi: 10.1016/j.gene.2022.147127 36563714

[B5] Aono A. H. Pimenta R. J. G. Garcia A. L. B. Correr F. H. Hosaka G. K. Carrasco M. M.. (2021). The wild sugarcane and sorghum kinomes: Insights into expansion, diversification, and expression patterns. Front. Plant Sci. 12, 668623. doi: 10.3389/fpls.2021.668623 34305969 PMC8294386

[B6] Badouin H. Gouzy J. Grassa C. J. Murat F. Staton S. E. Cottret L.. (2017). The sunflower genome provides insights into oil metabolism, flowering and Asterid evolution. Nature 546, 148–152. doi: 10.1038/nature22380 28538728

[B7] Cao S. Zheng Y. Wang K. Jin P. Rui H. (2009). Methyl jasmonate reduces chilling injury and enhances antioxidant enzyme activity in postharvest loquat fruit. Food Chem. 115, 1458–1463. doi: 10.1016/j.foodchem.2009.01.082

[B8] Chen C. Chen H. Zhang Y. Thomas H. R. Frank M. H. He Y.. (2020). TBtools: an integrative toolkit developed for interactive analyses of big biological data. Mol. Plant 13, 1194–1202. doi: 10.1016/j.molp.2020.06.009 32585190

[B9] Chu J. Monte I. Defalco T. A. Köster P. Derbyshire P. Menke F. L.. (2023). Conservation of the PBL-RBOH immune module in land plants. Curr. Biol. 33, 1130–1137.e5. doi: 10.1016/j.cub.2023.01.050 36796360

[B10] Cohen P. (2002). The origins of protein phosphorylation. Nat. Cell Biol. 4, E127–E130. doi: 10.1038/ncb0502-e127 11988757

[B11] Collin A. Daszkowska-Golec A. S zarejko I. (2021). Updates on the role of ABSCISIC ACID INSENSITIVE 5 (ABI5) and ABSCISIC ACID-RESPONSIVE ELEMENT BINDING FACTORs (ABFs) in ABA signaling in different developmental stages in plants. Cells 10, 1996. doi: 10.3390/cells10081996 34440762 PMC8394461

[B12] Costa A. T. Bravo J. P. Krause-Sakate R. Maia I. G. (2016). The receptor-like kinase SlSOBIR1 is differentially modulated by virus infection but its overexpression in tobacco has no significant impact on virus accumulation. Plant Cell Rep. 35, 65–75. doi: 10.1007/s00299-015-1868-8 26408145

[B13] Cui Y. Hu C. Zhu Y. Cheng K. Li X. Wei Z.. (2018). CIK receptor kinases determine cell fate specification during early anther development in Arabidopsis. Plant Cell 30, 2383–2401. doi: 10.1105/tpc.17.00586 30201822 PMC6241272

[B14] Defalco T. A. Zipfel C. (2021). Molecular mechanisms of early plant pattern-triggered immune signaling. Mol. Cell 81, 3449–3467. doi: 10.1016/j.molcel.2021.07.029 34403694

[B15] Dezső Z. Nikolsky Y. Sviridov E. Shi W. Serebriyskaya T. Dosymbekov D.. (2008). A comprehensive functional analysis of tissue specificity of human gene expression. BMC Biol. 6, 1–15. doi: 10.1186/1741-7007-6-49 19014478 PMC2645369

[B16] Diener A. C. Ausubel F. M. (2005). RESISTANCE TO FUSARIUM OXYSPORUM 1, a dominant Arabidopsis disease-resistance gene, is not race specific. Genetics 171, 305–321. doi: 10.1534/genetics.105.042218 15965251 PMC1456520

[B17] Ding Y. Li H. Zhang X. Xie Q. Gong Z. Yang S.. (2015). OST1 kinase modulates freezing tolerance by enhancing ICE1 stability in Arabidopsis. Dev. Cell 32, 278–289. doi: 10.1016/j.devcel.2014.12.023 25669882

[B18] Dodds P. N. Rathjen J. P. (2010). Plant immunity: towards an integrated view of plant–pathogen interactions. Nat. Rev. Genet. 11, 539–548. doi: 10.1038/nrg2812 20585331

[B19] Duriez P. Vautrin S. Auriac M.-C. Bazerque J. Boniface M.-C. Callot C.. (2019). A receptor-like kinase enhances sunflower resistance to Orobanche cumana. Nat. Plants 5, 1211–1215. doi: 10.1038/s41477-019-0556-z 31819219

[B20] Duvaud S. Gabella C. Lisacek F. Stockinger H. Ioannidis V. Durinx C.. (2021). Expasy, the Swiss Bioinformatics Resource Portal, as designed by its users. Nucleic Acids Res. 49, W216–W227. doi: 10.1093/nar/gkab225 33849055 PMC8265094

[B21] Escobar-Restrepo J.-M. Huck N. Kessler S. Gagliardini V. Gheyselinck J. Yang W.-C.. (2007). The FERONIA receptor-like kinase mediates male-female interactions during pollen tube reception. Science 317, 656–660. doi: 10.1126/science.1143562 17673660

[B22] Escocard De Azevedo Manhães A. M. Ortiz-Morea F. A. He P. Shan L. (2021). Plant plasma membrane-resident receptors: Surveillance for infections and coordination for growth and development. J. Integr. Plant Biol. 63, 79–101. doi: 10.1111/jipb.13051 33305880 PMC7855669

[B23] Fang Y. Xiong L. (2015). General mechanisms of drought response and their application in drought resistance improvement in plants. Cell. Mol. Life Sci. 72, 673–689. doi: 10.1007/s00018-014-1767-0 25336153 PMC11113132

[B24] Ferreira-Neto J. R. C. Borges A. N. D. C. Da Silva M. D. Morais D. D. L. Bezerra-Neto J. P. Bourque G.. (2021). The cowpea kinome: Genomic and transcriptomic analysis under biotic and abiotic stresses. Front. Plant Sci. 12, 667013. doi: 10.3389/fpls.2021.667013 34194450 PMC8238008

[B25] Finn R. D. Clements J. Eddy S. R. (2011). HMMER web server: interactive sequence similarity searching. Nucleic Acids Res. 39, W29–W37. doi: 10.1093/nar/gkr367 21593126 PMC3125773

[B26] Finn R. D. Mistry J. Tate J. Coggill P. Heger A. Pollington J. E.. (2010). The Pfam protein families database. Nucleic Acids Res. 38, D211–D222. doi: 10.1093/nar/gkp985 19920124 PMC2808889

[B27] Fischer E. H. Krebs E. G. (1955). Conversion of phosphorylase b to phosphorylase a in muscle extracts. J. Biol. Chem. 216, 121–132. doi: 10.1016/S0021-9258(19)52289-X 13252012

[B28] Freeling M. (2009). Bias in plant gene content following different sorts of duplication: tandem, whole-genome, segmental, or by transposition. Annu. Rev. Plant Biol. 60, 433–453. doi: 10.1146/annurev.arplant.043008.092122 19575588

[B29] Gigli-Bisceglia N. Van Zelm E. Huo W. Lamers J. Testerink C. (2022). Arabidopsis root responses to salinity depend on pectin modification and cell wall sensing. Development 149, dev200363. doi: 10.1242/dev.200363 35574987 PMC9270968

[B30] Gill R. A. Ali B. Yang S. Tong C. Islam F. Gill M. B.. (2017). Reduced glutathione mediates pheno-ultrastructure, kinome and transportome in chromium-induced Brassica napus L. Front. Plant Sci. 8, 2037. doi: 10.3389/fpls.2017.02037 29312362 PMC5732361

[B31] Gong Z. Xiong L. Shi H. Yang S. Herrera-Estrella L. R. Xu G.. (2020). Plant abiotic stress response and nutrient use efficiency. Sci. China Life Sci. 63, 635–674. doi: 10.1007/s11427-020-1683-x 32246404

[B32] Guo H. Li L. Ye H. Yu X. Algreen A. Yin Y. (2009a). Three related receptor-like kinases are required for optimal cell elongation in Arabidopsis thaliana. Proc. Natl. Acad. Sci. 106, 7648–7653. doi: 10.1073/pnas.0812346106 19383785 PMC2678668

[B33] Guo H. Ye H. Li L. Yin Y. (2009b). A family of receptor-like kinases are regulated by BES1 and involved in plant growth in Arabidopsis thaliana. Plant Signaling Behav. 4, 784–786. doi: 10.4161/psb.4.8.9231 PMC2801400 19820315

[B34] Häffner E. Karlovsky P. Splivallo R. Traczewska A. Diederichsen E. (2014). ERECTA, salicylic acid, abscisic acid, and jasmonic acid modulate quantitative disease resistance of Arabidopsis thaliana to Verticillium longisporum. BMC Plant Biol. 14, 1–16. doi: 10.1186/1471-2229-14-85 PMC4021371 24690463

[B35] Hallgren J. Tsirigos K. D. Pedersen M. D. Almagro Armenteros J. J. Marcatili P. Nielsen H.. (2022). DeepTMHMM predicts alpha and beta transmembrane proteins using deep neural networks. BioRxiv 2022, 04.08.487609. doi: 10.1101/2022.04.08.487609

[B36] Holzberg S. Brosio P. Gross C. Pogue G. P. (2002). Barley stripe mosaic virus-induced gene silencing in a monocot plant. Plant J. 30, 315–327. doi: 10.1046/j.1365-313X.2002.01291.x 12000679

[B37] Hua D. Wang C. He J. Liao H. Duan Y. Zhu Z.. (2012). A plasma membrane receptor kinase, GHR1, mediates abscisic acid-and hydrogen peroxide-regulated stomatal movement in Arabidopsis. Plant Cell 24, 2546–2561. doi: 10.1105/tpc.112.100107 22730405 PMC3406912

[B38] Hurni S. Scheuermann D. Krattinger S. G. Kessel B. Wicker T. Herren G.. (2015). The maize disease resistance gene Htn1 against northern corn leaf blight encodes a wall-associated receptor-like kinase. Proc. Natl. Acad. Sci. 112, 8780–8785. doi: 10.1073/pnas.1502522112 26124097 PMC4507197

[B39] Janitza P. Ullrich K. K. Quint M. (2012). Toward a comprehensive phylogenetic reconstruction of the evolutionary history of mitogen-activated protein kinases in the plant kingdom. Front. Plant Sci. 3, 271. doi: 10.3389/fpls.2012.00271 23230446 PMC3515877

[B40] Krebs E. G. Fischer E. H. (1955). Phosphorylase activity of skeletal muscle extracts. J. Biol. Chem. 216, 113–120. doi: 10.1016/S0021-9258(19)52288-8 13252011

[B41] Labbé J. Muchero W. Czarnecki O. Wang J. Wang X. Bryan A. C.. (2019). Mediation of plant–mycorrhizal interaction by a lectin receptor-like kinase. Nat. Plants 5, 676–680. doi: 10.1038/s41477-019-0469-x 31285560

[B42] Larkin M. A. Blackshields G. Brown N. P. Chenna R. Mcgettigan P. A. Mcwilliam H.. (2007). Clustal W and clustal X version 2.0. bioinformatics 23, 2947–2948. doi: 10.1093/bioinformatics/btm404 17846036

[B43] Lehti-Shiu M. D. Zou C. Hanada K. Shiu S-H. (2009). Evolutionary history and stress regulation of plant receptor-like kinase/pelle genes. Plant Physiol. 150, 12–26. doi: 10.1104/pp.108.134353 19321712 PMC2675737

[B44] Li B. Ferreira M. A. Huang M. Camargos L. F. Yu X. Teixeira R. M.. (2019). The receptor-like kinase NIK1 targets FLS2/BAK1 immune complex and inversely modulates antiviral and antibacterial immunity. Nat. Commun. 10, 4996. doi: 10.1038/s41467-019-12847-6 31676803 PMC6825196

[B45] Liu J. Chen N. Grant J. N. Cheng Z-M. Stewart C. N. Jr Hewezi T. (2015). Soybean kinome: functional classification and gene expression patterns. J. Exp. Bot. 66, 1919–1934. doi: 10.1093/jxb/eru537 25614662 PMC4378628

[B46] Liu H. Qu W. Zhu K. Cheng Z.-M. M. (2020). The wild strawberry kinome: identification, classification and transcript profiling of protein kinases during development and in response to gray mold infection. BMC Genomics 21, 1–14. doi: 10.1186/s12864-020-07053-4 PMC7490889 32928117

[B47] Lu S. Wang J. Chitsaz F. Derbyshire M. K. Geer R. C. Gonzales N. R.. (2020). CDD/SPARCLE: the conserved domain database in 2020. Nucleic Acids Res. 48, D265–D268. doi: 10.1093/nar/gkz991 31777944 PMC6943070

[B48] Lynch M. Conery J. S. (2000). The evolutionary fate and consequences of duplicate genes. science 290, 1151–1155. doi: 10.1126/science.290.5494.1151 11073452

[B49] Ma X. Gai W.-X. Li Y. Yu Y.-N. Ali M. Gong Z.-H. (2022). The CBL-interacting protein kinase CaCIPK13 positively regulates defence mechanisms against cold stress in pepper. J. Exp. Bot. 73, 1655–1667. doi: 10.1093/jxb/erab505 35137060

[B50] Mishra D. Suri G. S. Kaur G. Tiwari M. (2021). Comprehensive analysis of structural, functional, and evolutionary dynamics of Leucine Rich Repeats-RLKs in Thinopyrum elongatum. Int. J. Biol. Macromolecules 183, 513–527. doi: 10.1016/j.ijbiomac.2021.04.137 33933540

[B51] Miyazaki S. Murata T. Sakurai-Ozato N. Kubo M. Demura T. Fukuda H.. (2009). ANXUR1 and 2, sister genes to FERONIA/SIRENE, are male factors for coordinated fertilization. Curr. Biol. 19, 1327–1331. doi: 10.1016/j.cub.2009.06.064 19646876

[B52] Mohamed H. I. Latif H. H. (2017). Improvement of drought tolerance of soybean plants by using methyl jasmonate. Physiol. Mol. Biol. Plants 23, 545–556. doi: 10.1007/s12298-017-0451-x 28878493 PMC5567712

[B53] Nawaz M. Sun J. Shabbir S. Khattak W. A. Ren G. Nie X.. (2023). A review of plants strategies to resist biotic and abiotic environmental stressors. Sci. Total Environ. 900, 165832. doi: 10.1016/j.scitotenv.2023.165832 37524179

[B54] Owji H. Nezafat N. Negahdaripour M. Hajiebrahimi A. Ghasemi Y. (2018). A comprehensive review of signal peptides: Structure, roles, and applications. Eur. J. Cell Biol. 97, 422–441. doi: 10.1016/j.ejcb.2018.06.003 29958716

[B55] Price M. N. Dehal P. S. (2010). Arkin A P.FastTree 2–approximately maximum-likelihood trees for large alignments. PloS One 5, e9490. doi: 10.1371/journal.pone.0009490 20224823 PMC2835736

[B56] Qi J. Wang J. Gong Z. Zhou J.-M. (2017). Apoplastic ROS signaling in plant immunity. Curr. Opin. Plant Biol. 38, 92–100. doi: 10.1016/j.pbi.2017.04.022 28511115

[B57] Qiao X. Yin H. Li L. Wang R. Wu J. Wu J.. (2018). Different modes of gene duplication show divergent evolutionary patterns and contribute differently to the expansion of gene families involved in important fruit traits in pear (Pyrus bretschneideri). Front. Plant Sci. 9, 161. doi: 10.3389/fpls.2018.00161 29487610 PMC5816897

[B58] Santos L. B. Aono A. H. Francisco F. R. Silva C. C. Souza L. M. De Souza A. P. (2022). The rubber tree kinome: genome-wide characterization and insights into coexpression patterns associated with abiotic stress responses. bioRxiv 2022, 08. 24.505065. doi: 10.1101/2022.08.24.505065 PMC9941580 36824205

[B59] Schulze-Muth P. Irmler S. Schröder G. Schröder J. (1996). Novel type of receptor-like protein kinase from a higher plant (Catharanthus roseus): cDNA, gene, intramolecular autophosphorylation, and identification of a threonine important for auto-and substrate phosphorylation. J. Biol. Chem. 271, 26684–26689. doi: 10.1074/jbc.271.43.26684 8900145

[B60] Shahzad A. Pitann B. Ali H. Qayyum M. Fatima A. Bakhat H. (2015). Maize genotypes differing in salt resistance vary in jasmonic acid accumulation during the first phase of salt stress. J. Agron. Crop Sci. 201, 443–451. doi: 10.1111/jac.2015.201.issue-6

[B61] Shiu S.-H. Bleecker A. B. (2001). Receptor-like kinases from Arabidopsis form a monophyletic gene family related to animal receptor kinases. Proc. Natl. Acad. Sci. 98, 10763–10768. doi: 10.1073/pnas.181141598 11526204 PMC58549

[B62] Shiu S.-H. Karlowski W. M. Pan R. Tzeng Y.-H. Mayer K. F. Li W.-H. (2004). Comparative analysis of the receptor-like kinase family in Arabidopsis and rice. Plant Cell 16, 1220–1234. doi: 10.1105/tpc.020834 15105442 PMC423211

[B63] Singh D. K. Calviño M. Brauer E. K. Fernandez-Pozo N. Strickler S. Yalamanchili R.. (2014). The tomato kinome and the tomato kinase library ORFeome: novel resources for the study of kinases and signal transduction in tomato and solanaceae species. Mol. Plant-Microbe Interact. 27, 7–17. doi: 10.1094/MPMI-08-13-0218-TA 24047240

[B64] Soltabayeva A. Dauletova N. Serik S. Sandybek M. Omondi J. O. Kurmanbayeva A.. (2022). Receptor-like Kinases (LRR-RLKs) in response of plants to biotic and abiotic stresses. Plants 11, 2660. doi: 10.3390/plants11192660 36235526 PMC9572924

[B65] Strasburg J. L. Rieseberg L. H. (2008). Molecular demographic history of the annual sunflowers Helianthus annuus and H. petiolaris—large effective population sizes and rates of long-term gene flow. Evolution 62, 1936–1950. doi: 10.1111/j.1558-5646.2008.00415.x 18462213 PMC2601659

[B66] Sun P. Jiao B. Yang Y. Shan L. Li T. Li X.. (2022). WGDI: A user-friendly toolkit for evolutionary analyses of whole-genome duplications and ancestral karyotypes. Mol. Plant 15, 1841–1851. doi: 10.1016/j.molp.2022.10.018 36307977

[B67] Tamura K. Stecher G. (2021). Kumar S.MEGA11: molecular evolutionary genetics analysis version 11. Mol. Biol. Evol. 38, 3022–3027. doi: 10.1093/molbev/msab120 33892491 PMC8233496

[B68] Tang D. Wang G. Zhou J. M. (2017). Receptor kinases in plant-pathogen interactions: more than pattern recognition. Plant Cell 29, 618–637. doi: 10.1105/tpc.16.00891 28302675 PMC5435430

[B69] Trinh N. N. Huang T. L. Chi W. C. Fu S. F. Chen C. C. Huang H. J. (2014). Chromium stress response effect on signal transduction and expression of signaling genes in rice. Physiologia plantarum 150, 205–224. doi: 10.1111/ppl.2014.150.issue-2 24033343

[B70] Wagner T. A. Kohorn B. D. (2001). Wall-associated kinases are expressed throughout plant development and are required for cell expansion. Plant Cell 13, 303–318. doi: 10.1105/tpc.13.2.303 11226187 PMC102244

[B71] Wan J. Patel A. Mathieu M. Kim S.-Y. Xu D. Stacey G. (2008). A lectin receptor-like kinase is required for pollen development in Arabidopsis. Plant Mol. Biol. 67, 469–482. doi: 10.1007/s11103-008-9332-6 18392777

[B72] Wang Y. Cordewener J. H. America A. H. Shan W. Bouwmeester K. Govers F. (2015). Arabidopsis lectin receptor kinases LecRK-IX. 1 and LecRK-IX. 2 are functional analogs in regulating Phytophthora resistance and plant cell death. Mol. Plant-Microbe Interact. 28, 1032–1048. doi: 10.1094/MPMI-02-15-0025-R 26011556

[B73] Wang Y. Tang H. Debarry J. D. Tan X. Li J. Wang X.. (2012). MCScanX: a toolkit for detection and evolutionary analysis of gene synteny and collinearity. Nucleic Acids Res. 40, e49–e49. doi: 10.1093/nar/gkr1293 22217600 PMC3326336

[B74] Wei K. Li Y. (2019). Functional genomics of the protein kinase superfamily from wheat. Mol. Breed. 39, 1–23. doi: 10.1007/s11032-019-1045-9

[B75] Wei K. Wang Y. Xie D. (2014). Identification and expression profile analysis of the protein kinase gene superfamily in maize development. Mol. Breed. 33, 155–172. doi: 10.1007/s11032-013-9941-x

[B76] Yan J. Li G. Guo X. Li Y. Cao X. (2018). Genome-wide classification, evolutionary analysis and gene expression patterns of the kinome in Gossypium. PloS One 13, e0197392. doi: 10.1371/journal.pone.0197392 29768506 PMC5955557

[B77] Yan J. Su P. Wei Z. Nevo E. Kong L. (2017). Genome-wide identification, classification, evolutionary analysis and gene expression patterns of the protein kinase gene family in wheat and Aegilops tauschii. Plant Mol. Biol. 95, 227–242. doi: 10.1007/s11103-017-0637-1 28918554

[B78] Yang C. Yi-Feng J. Yushu W. Yansong G. Qi W. Xue Y. (2022). Diverse roles of the CIPK gene family in transcription regulation and various biotic and abiotic stresses: A literature review and bibliometric study. Front. Genet. 13, 1041078. doi: 10.3389/fgene.2022.1041078 36457742 PMC9705351

[B79] Yue J.-Y. Jiao J.-L. Wang W.-W. Jie X.-R. Wang H.-Z. (2023). Silencing of the calcium-dependent protein kinase TaCDPK27 improves wheat resistance to powdery mildew. BMC Plant Biol. 23, 1–11. doi: 10.1186/s12870-023-04140-y 36882703 PMC9993671

[B80] Zhang H. Zhu J. Gong Z. Zhu J.-K. (2022). Abiotic stress responses in plants. Nat. Rev. Genet. 23, 104–119. doi: 10.1038/s41576-021-00413-0 34561623

[B81] Zhao Y. Du H. Wang Y. Wang H. Yang S. Li C.. (2021). The calcium-dependent protein kinase ZmCDPK7 functions in heat-stress tolerance in maize. J. Integr. Plant Biol. 63, 510–527. doi: 10.1111/jipb.13056 33331695

[B82] Zheng Y. Jiao C. Sun H. Rosli H. G. Pombo M. A. Zhang P.. (2016). iTAK: a program for genome-wide prediction and classification of plant transcription factors, transcriptional regulators, and protein kinases. Mol. Plant 9, 1667–1670. doi: 10.1016/j.molp.2016.09.014 27717919

[B83] Zhou Y.-B. Liu C. Tang D.-Y. Yan L. Wang D. Yang Y.-Z.. (2018). The receptor-like cytoplasmic kinase STRK1 phosphorylates and activates CatC, thereby regulating H2O2 homeostasis and improving salt tolerance in rice. Plant Cell 30, 1100–1118. doi: 10.1105/tpc.17.01000 29581216 PMC6002193

[B84] Zhu T. Deng X. Zhou X. Zhu L. Zou L. Li P.. (2016). Ethylene and hydrogen peroxide are involved in brassinosteroid-induced salt tolerance in tomato. Sci. Rep. 6, 35392. doi: 10.1038/srep35392 27739520 PMC5064326

[B85] Zhu Q. Feng Y. Xue J. Chen P. Zhang A. Yu Y. (2023). Advances in receptor-like protein kinases in balancing plant growth and stress responses. Plants 12, 427. doi: 10.3390/plants12030427 36771514 PMC9919196

[B86] Zhu K. Liu H. Chen X. Cheng Q. Cheng Z.-M. M. (2018a). The kinome of pineapple: catalog and insights into functions in crassulacean acid metabolism plants. BMC Plant Biol. 18, 1–16. doi: 10.1186/s12870-018-1389-z 30227850 PMC6145126

[B87] Zhu K. Wang X. Liu J. Tang J. Cheng Q. Chen J.-G.. (2018b). The grapevine kinome: annotation, classification and expression patterns in developmental processes and stress responses. Horticulture Res. 5, 19. doi: 10.1038/s41438-018-0027-0 PMC5878832 29619230

[B88] Zulawski M. Schulze G. Braginets R. Hartmann S. Schulze W. X. (2014). The Arabidopsis Kinome: phylogeny and evolutionary insights into functional diversification. BMC Genomics 15, 1–15. doi: 10.1186/1471-2164-15-548 24984858 PMC4112214

[B89] Zuo W. Chao Q. Zhang N. Ye J. Tan G. Li B.. (2015). A maize wall-associated kinase confers quantitative resistance to head smut. Nature Genet. 47, 151–157. doi: 10.1038/ng.3170 25531751

