# Supplementary figures and images for: Genome-wide characterization of the sunflower kinome: classification, evolutionary analysis and expression patterns under different stresses
Source: Front Plant Sci. 2024 Dec 2;15:1450936. doi: 10.3389/fpls.2024.1450936 (PMC11646777; doi:10.3389/fpls.2024.1450936)

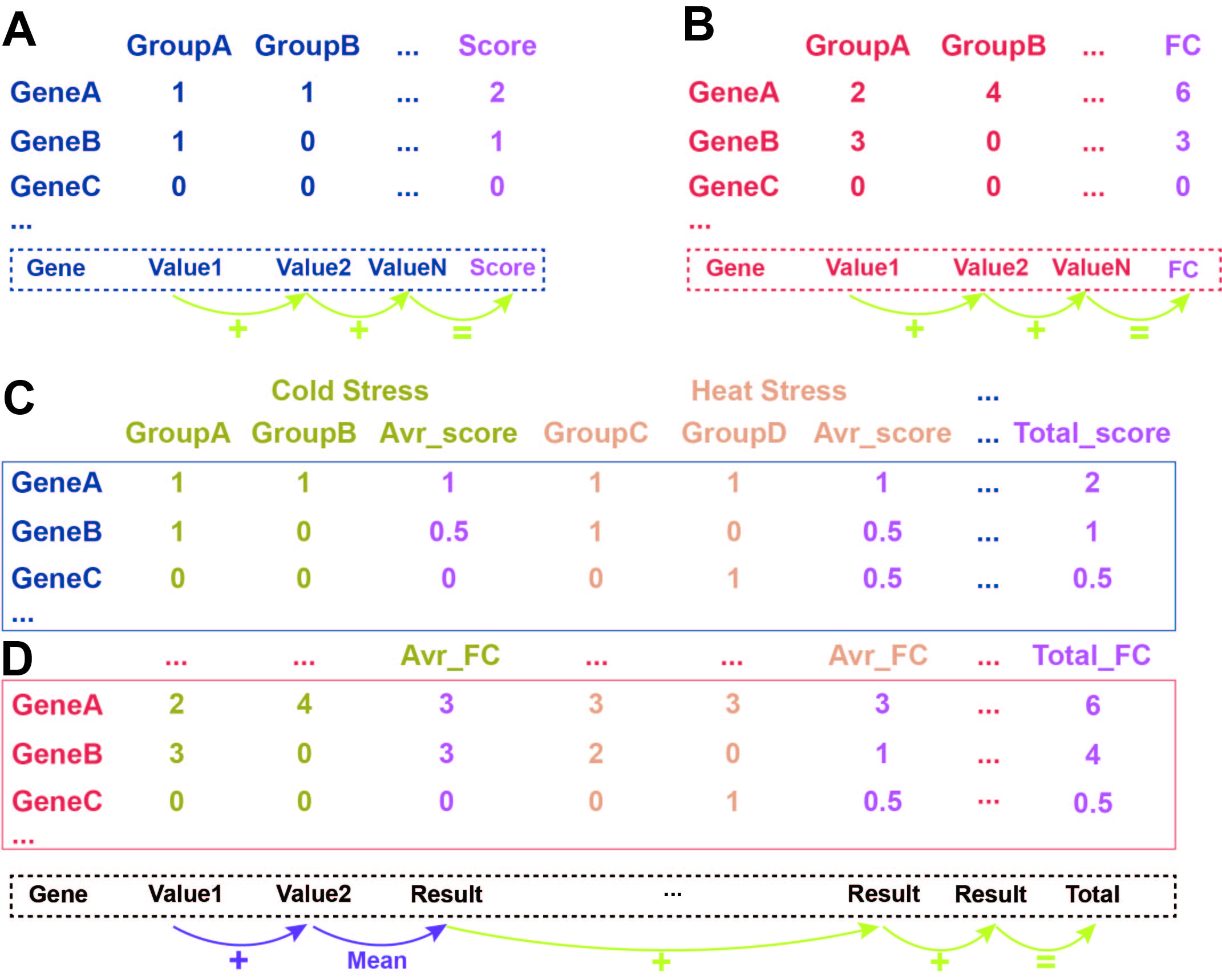

Supplement: Supplementary Figure 1 — Scoring method of sunflower protein kinase under different biological and abiotic stresses. FC, Fold Change. [file Image1.jpeg]

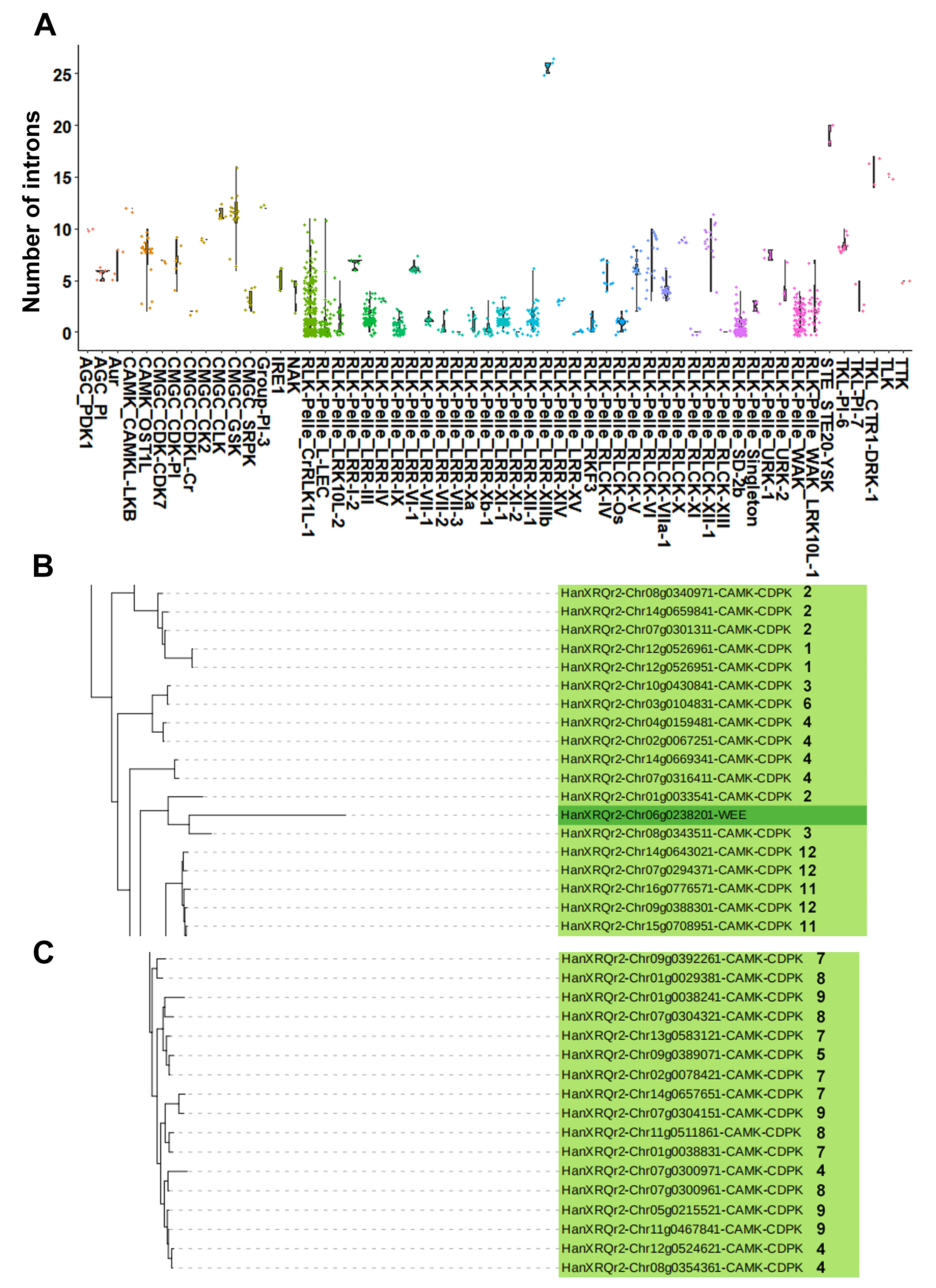

Supplement: Supplementary Figure 3 — The distribution trend of protein kinase introns within subfamilies and its relationship with genetic distances in the phylogenetic tree. (A) Distribution trend of protein kinases introns within subfamilies. (B, C) The relationship between the number of introns in protein kinases and the branches they occupy in the phylogenetic tree. [file Image3.jpeg]

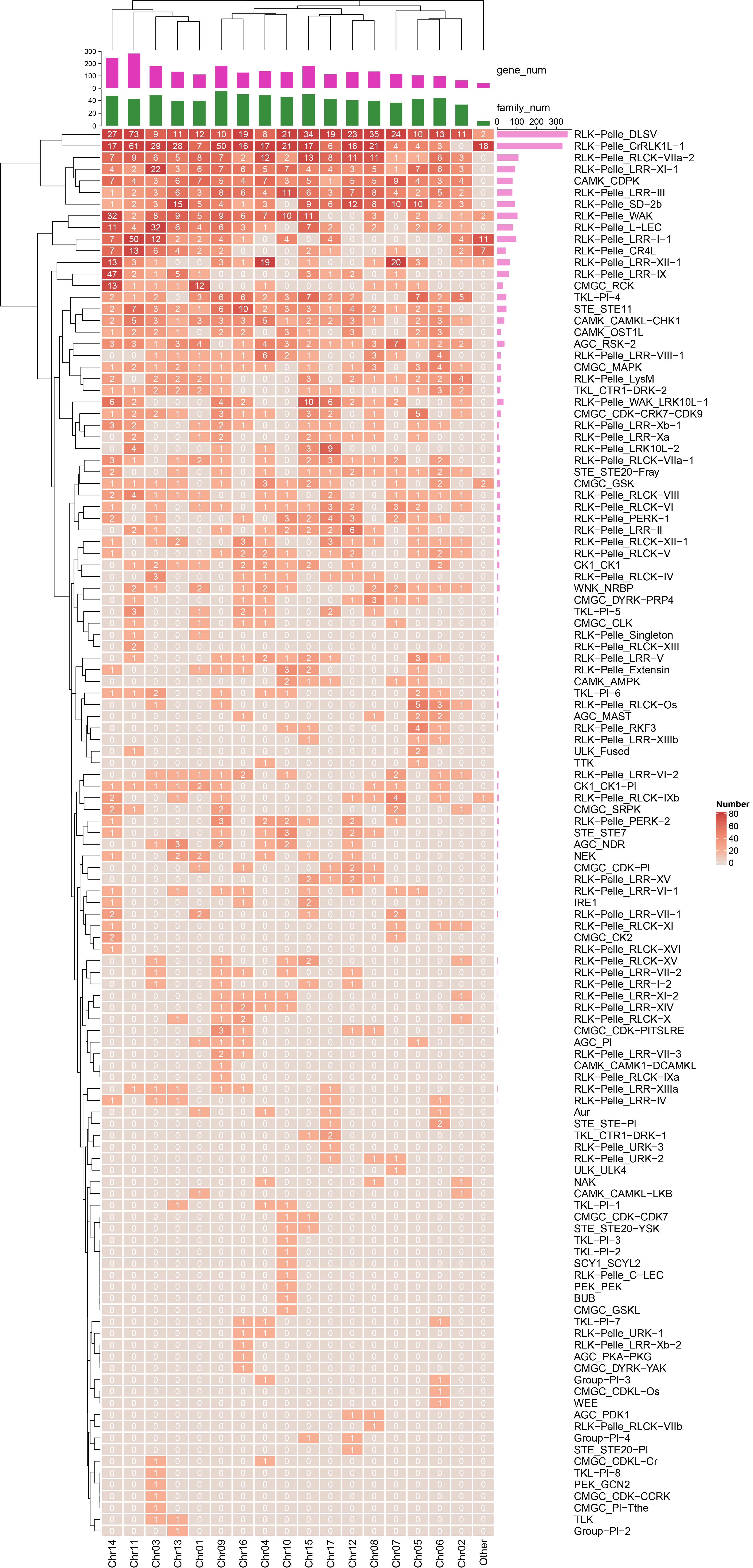

Supplement: Supplementary Figure 4 — Chromosomal distribution of sunflower protein kinase family. [file Image4.jpeg]

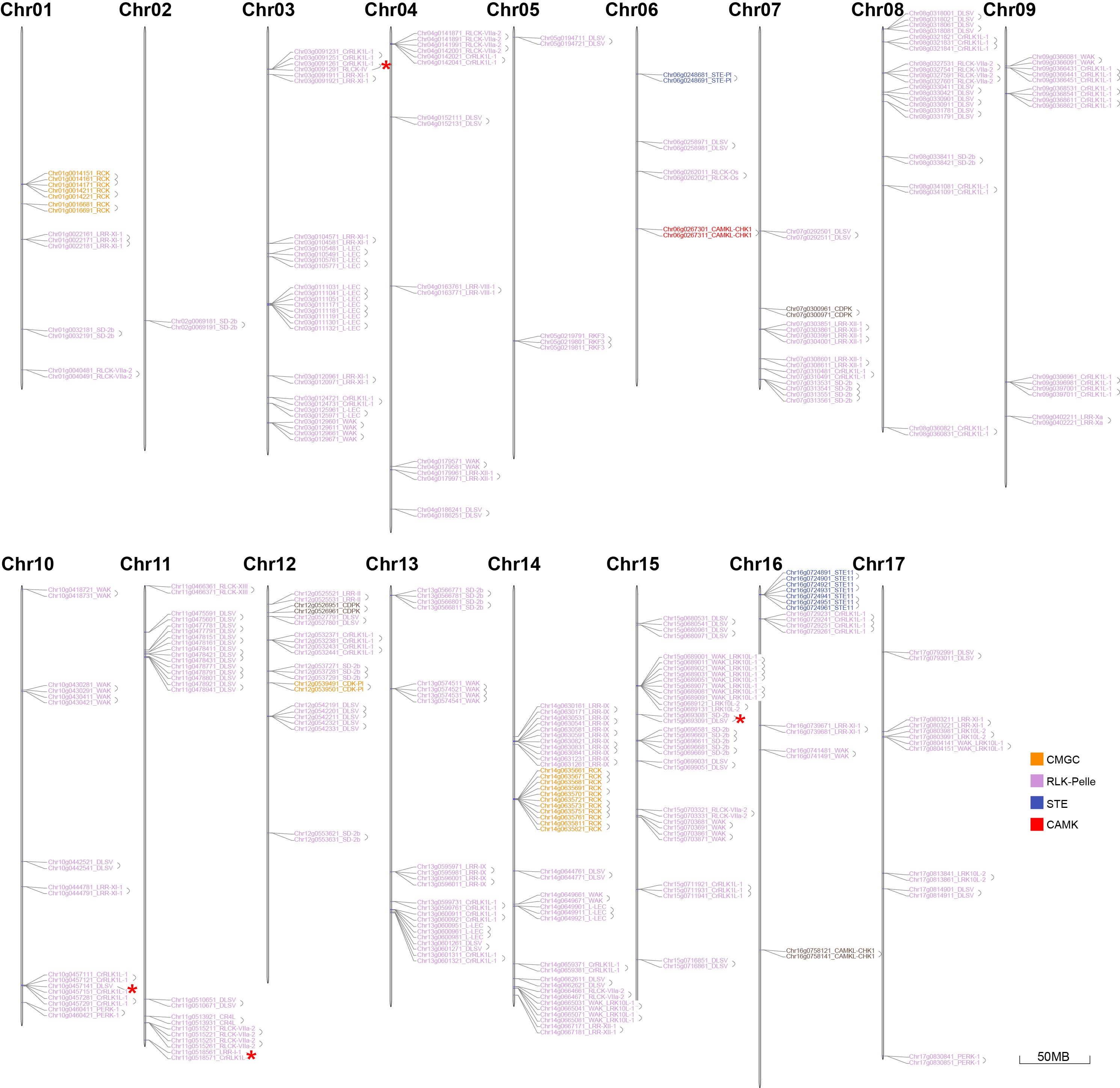

Supplement: Supplementary Figure 5 — Chromosomal locations of the 166 tandemly arrayed sunflower PK gene pairs. This figure uses different colors to indicate different gene families. [file Image5.jpeg]

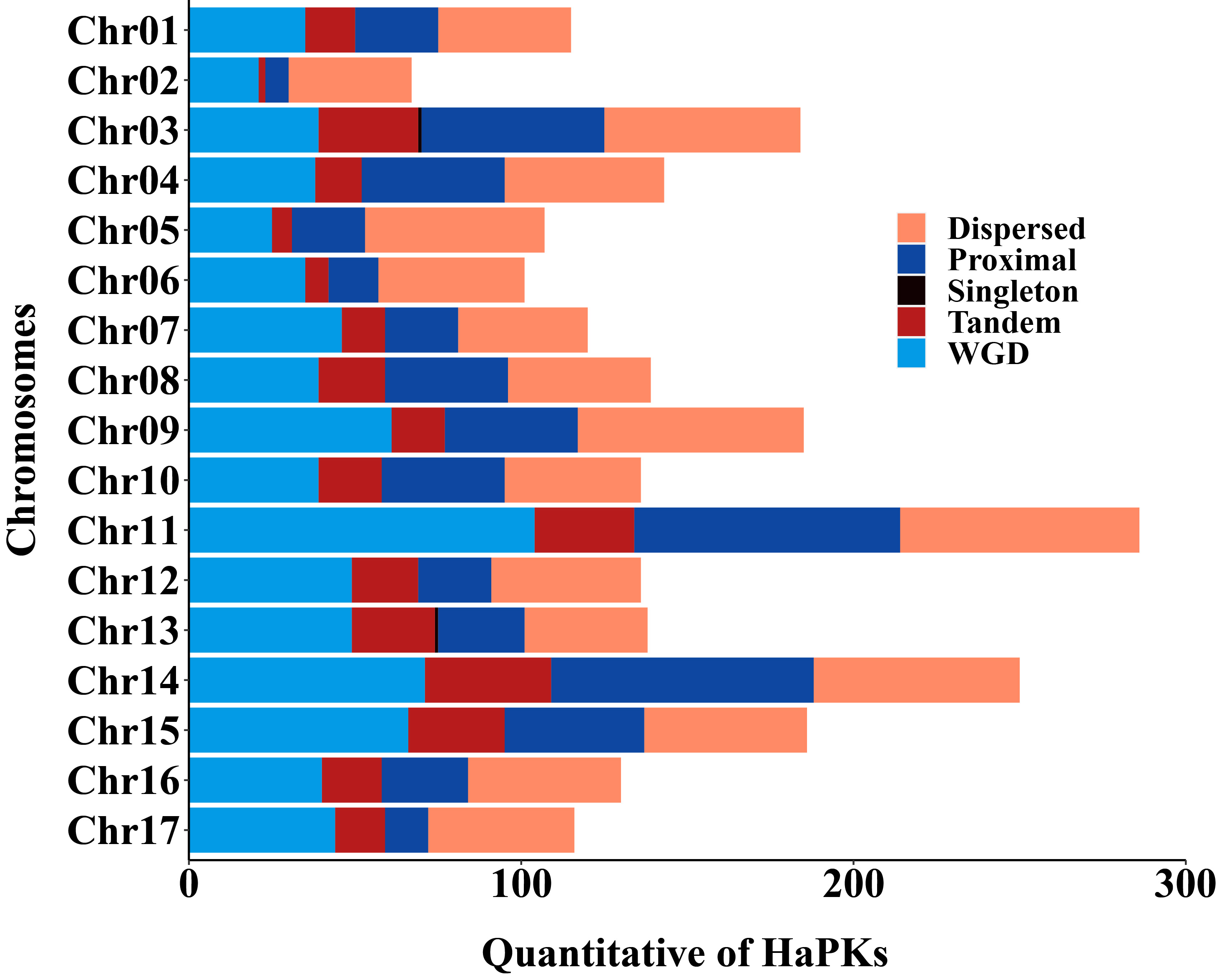

Supplement: Supplementary Figure 6 — The distribution trend of protein kinase duplication events on chromosomes. [file Image6.jpeg]
